# Integrated single-cell multiomics reveals neutrophil-driven immune-metabolic reprogramming of Kupffer cells via thrombospondin-1/CD36 after surgical stress

**DOI:** 10.1172/jci.insight.203495

**Published:** 2026-09-22

**Authors:** Hongji Zhang, Xingping Huang, Preethi Jayakumar, Yunwei Zhang, Ti Yang, Xiaorong Guo, Grace Wu, Amrendra Kumar, Vijaya Bharti, Amblessed Onuma, Chengli Shen, Dequan Lou, Patricia S. Latham, James M. Crawford, Kong Chen, Anna E. Vilgelm, Lopa Mishra, Allan Tsung, Meihong Deng, Hai Huang

**Affiliations:** 1 Department of Surgery, School of Medicine, University of Virginia, Charlottesville, Virginia, USA.; 2 Center for Immunology and Inflammation, Institute of Translational Research, Feinstein Institutes for Medical Research, Manhasset, New York, USA.; 3 Department of Surgery, Northwell Health, Manhasset, New York, USA.; 4 Department of Pathology, The Ohio State University, Columbus, Ohio, USA.; 5 Department of Surgical Oncology, MD Anderson Cancer Center, Houston, Texas, USA.; 6 Department of Medicine, University of Pittsburgh, Pittsburgh, Pennsylvania, USA.; 7 Department of Pathology, George Washington University, Washington, DC, USA.; 8 Department of Pathology and Laboratory Medicine, Northwell Health, Manhasset, New York, USA.; 9 Institute for Bioelectronic Medicine, Feinstein Institutes for Medical Research and Cold Spring Harbor Laboratory, Department of Medicine, Division of Gastroenterology and Hepatology, Northwell Health, Manhasset, New York, USA.; 10 Division of Hepatology, Department of Medicine, Northwell Health, Manhasset, New York, USA.

**Keywords:** Hepatology, Immunology, Inflammation, Adaptive immunity, Innate immunity, Surgery

## Abstract

Surgical stress, such as liver ischemia/reperfusion (I/R) injury characterized by robust neutrophil infiltration and immune activation, induces sterile inflammation that reshapes tissue immunity and contributes to organ dysfunction, yet the intercellular circuits that spatially orchestrate these responses within the hepatic immune microenvironment remain incompletely defined. Here, we integrate single-cell RNA sequencing, spatial transcriptomics, high-dimensional spectral flow cytometry, and metabolomics to resolve the hepatic immune landscape following I/R at cellular and spatial resolution. While confirming extensive immune remodeling, we identify a dominant neutrophil–Kupffer cell communication axis mediated by neutrophil-derived thrombospondin-1 (TSP-1), which selectively engages CD36 on Kupffer cells. This interaction drives coordinated immune-metabolic reprogramming in Kupffer cells, characterized by suppression of oxidative phosphorylation, enhanced glycolysis, and remodeling of sphingolipid metabolism, including accumulation of hexosylceramides. Spatial analyses reveal preferential neutrophil–Kupffer cell colocalization within necrotic niches, and cross-species integration with human liver transplant datasets demonstrates conserved upregulation of the TSP-1/CD36 axis following reperfusion. Pharmacologic inhibition of TSP-1 attenuates liver injury and inflammatory responses in vivo. Together, these findings define a spatially organized, neutrophil-driven immune-metabolic circuit that governs functional reprogramming of Kupffer cells during surgical stress and identify the TSP-1/CD36 pathway as a conserved and targetable mediator of sterile liver injury.

## Abstract 2

We identified how immune cells communicate after liver surgery and uncovered a potential treatment target to reduce inflammation and liver damage.

## Introduction

Surgical stress, defined by cellular and organ system insult, tissue injury, and nociceptive stimulation during surgical procedures, triggers a cascade of neurohormonal and immune responses. Although initially protective, these responses often become dysregulated, leading to excessive inflammation, metabolic imbalance, and tissue injury (1, 2). Liver ischemia/reperfusion (I/R) is an inevitable surgical stress during major liver resection or liver transplantation (3). The resulting local and systemic inflammation substantially contributes to postoperative morbidity, mortality, and health care burden (4, 5). However, the precise cellular circuits and intercellular interactions that orchestrate I/R–induced hepatic inflammation and injury remain incompletely understood.

The liver is a highly specialized immunological organ enriched with diverse resident and infiltrating immune populations whose functional states, spatial organization interactions, and metabolic reprogramming shape the inflammatory landscape of the hepatic immune microenvironment (6, 7). During I/R, both resident macrophages (Kupffer cells [KCs]) and recruited myeloid cells rapidly respond to injury, yet how these populations spatially coordinate and metabolically adapt within the hepatic microenvironment remains unclear. As immune cell function is tightly linked to metabolic state, resolving immune-metabolic interactions at cellular resolution is critical for understanding the pathogenesis of hepatic I/R–induced injury.

Recent advances in single-cell RNA sequencing (scRNA-seq) (8), spatial transcriptomics (9, 10), high-dimensional spectral flow cytometry (11, 12), and metabolomics (8, 13) have enabled high-resolution characterization of the hepatic immune microenvironment. Multiomics approaches integrating scRNA-seq, spatial transcriptomics, high-dimensional cytometry, and metabolomics provide complementary insights into cellular identity, spatial localization, and metabolic state. Although such approaches have been applied to characterize immune heterogeneity in steady-state and chronic liver diseases, a comprehensive, spatially resolved immune-metabolic map of hepatic I/R injury is lacking.

Here, we applied an integrated single-cell multiomics strategy to dissect the hepatic immune microenvironment in a well-established preclinical mouse model of liver I/R (14). We define dynamic remodeling of resident and infiltrating immune populations after I/R. Our analyses reveal marked neutrophil infiltration into necrotic zones and pronounced metabolic reprogramming of KCs. Integrative cell-cell interaction modeling identifies thrombospondin-1–mediated (TSP-1–mediated) signaling as a dominant neutrophil-derived pathway engaging CD36 on KCs, linking spatial immune crosstalk to metabolic rewiring and liver injury. Cross-species validation using human liver transplantation datasets further supports conservation of this immune-metabolic axis. Together, our findings establish a spatially organized neutrophil-KC circuit that drives sterile liver inflammation during surgical stress.

## Results

### Integrated single-cell multiomics reveals spatially organized immune remodeling after liver I/R.

**Figure 1:**
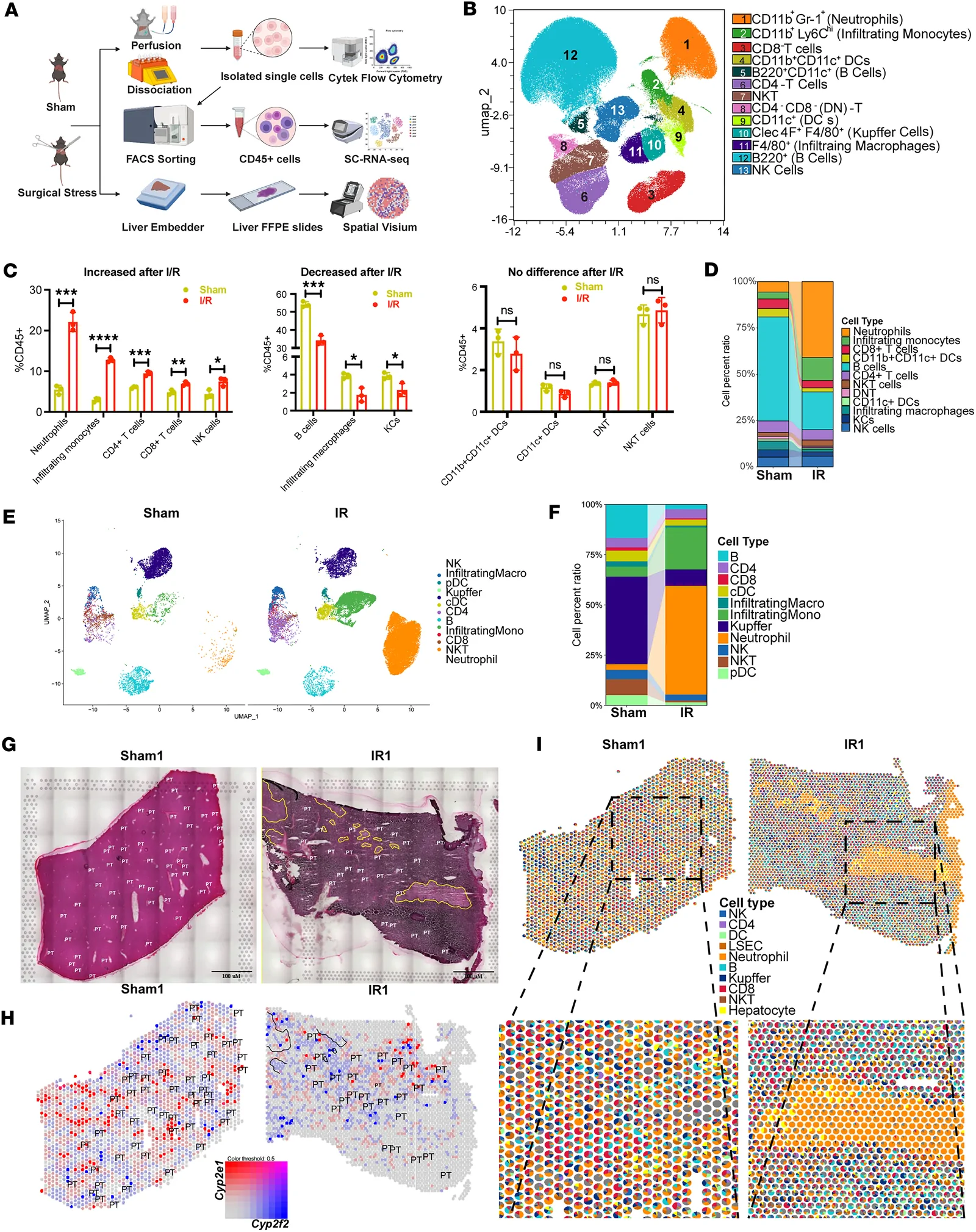
A single-cell multiomics atlas of murine liver before and after liver I/R. (**A**) Schematic diagram illustrating the single-cell multiomics atlas of murine liver before and after liver I/R. (**B**) Global UMAP of FlowSOM data derived from FACS-sorted CD45^+^ hepatic immune cells. (**C**) Quantification of hepatic immune cell populations showing significantly increased, decreased, or unchanged subsets following I/R. (**D**) Stacked bar graph depicting the proportions of hepatic immune cell populations in sham and I/R livers. (**E**) UMAP of scRNA-seq data showing CD45^+^ immune cell types extracted from the global UMAP space in sham and I/R samples. (**F**) Stacked bar graph showing the proportional distribution of immune cell types in sham and I/R livers. (**G**) Representative histological zonation of liver sections from sham and I/R mice used for spatial transcriptomics. Portal tracts (PT) are indicated, with the base of the “PT” label marking the precise location of the tract. Scale bars: 100 μm. (**H**) Identification of liver zonation in spatial transcriptomics specimens based on expression of *Cyp2f2* and *Cyp2e1*. (**I**) Spatial scatter-pie plot in which each spot is represented as a pie chart indicating the predicted proportion of constituent cell types. Data represent the mean ± SEM. *P < 0.05, **P < 0.01, ***P < 0.001, ****P < 0.0001, by 2-tailed, unpaired *t* test (**C**).

To define how surgical stress reshapes the hepatic immune microenvironment, we applied an integrated multiomics strategy combining high-dimensional spectral flow cytometry, scRNA-seq, and spatial transcriptomics in a murine model (8) of liver I/R injury (Figure 1A). As expected, I/R resulted in marked hepatocellular injury, evidenced by elevated serum alanine aminotransferase and extensive necrosis (Supplemental Figure 1, A and B; supplemental material available online with this article; https://doi.org/10.1172/jci.insight.203495DS1).

High-dimensional spectral flow cytometry of CD45^+^ hepatic immune cells stained with a comprehensive immune marker panel (15) revealed substantial remodeling of the immune landscape following I/R. Based on a multivariate *t*-mixture model and FlowSOM (16) clustering algorithm and clustered heatmap analysis, unbiased clustering identified major myeloid and lymphoid populations (Figure 1B and Supplemental Figure 1, C–E). Quantitative analysis demonstrated a pronounced expansion of CD11b^+^Gr-1^+^ neutrophils, Ly6C^+^ monocytes, NK cells, h CD4^+^ and CD8^+^ T cells after I/R, accompanied by a significant reduction in B cells and macrophages, including infiltrating macrophages and hepatic resident macrophages, Kupffer cells (KCs) (Figure 1, C and D). In contrast, dendritic cell and NKT cell numbers were unchanged after liver I/R.

To resolve transcriptional changes at single-cell resolution, we integrated newly generated scRNA-seq data with our previously published scRNA-seq dataset (Gene Expression Omnibus GSE173429, ref. 8) with newly performed animal experiments using the Harmony algorithm (17) to minimize experimental batch effects. In addition, to avoid unexpected noise and expression artifacts by dissociation, ribosomal genes (1,253 genes) were controlled and excluded (18). All 38,317 cells were clustered into 32 discrete clusters (Supplemental Figure 2, A and B). After qualitative filtering and exclusion of cells negative for *Ptprc* (CD45) (liver sinusoidal endothelial cells [LSECs], *n* = 4,067, clusters 9, 14, and 15; and hepatocytes, *n* = 264, cluster 28), 33,986 CD45^+^ immune cells were analyzed and classified into major immune subsets based on established feature genes (8): KCs, conventional dendritic cells (cDCs), plasmacytoid dendritic cells (pDCs), infiltrating monocytes, infiltrating macrophages, neutrophils, B cells, CD3^+^ T cells (CD4^+^ and CD8^+^ T cells), NK cells, and NKT cells (Figure 1E and Supplemental Figure 2C). Consistent with our flow cytometry findings, neutrophils and infiltrating monocytes represented the most prominently expanded populations after I/R (Figure 1F). The proportion of all other immune cells decreased, indicating that I/R injury not only caused immune cell infiltration into the liver but also modulated the transcriptomic landscape of the hepatic immune microenvironment (Figure 1F and Supplemental Figure 2D).

Given the highly zonated architecture of the liver, we next used spatial transcriptomics (ST) (19) to determine how immune cells redistribute within injured tissue. Visium-based (Visium Spatial, 10x Genomics) (20) profiling of sham and I/R liver sections (*n* = 2 per group) enabled spatial mapping of transcriptomic signatures across lobular zones (Figure 1, G and H, and Supplemental Figure 2E). To identify the zonation of the ST specimen, we used the specific expression of well-defined zonation markers, *Cyp2f2* and *Cyp2e1* (10), to indicate the periportal zone and pericentral zone, respectively (Figure 1H). Our ST zonation showed results consistent with histological analysis on hematoxylin and eosin (H&E) staining slides, comparing favorably with inferred zonation based on localization of portal tracts on the H&E section images.

We further integrated our annotated scRNA-seq dataset to sequence the transcriptome of FACS-CD45^+^-selected hepatic leukocytes from sham versus I/R mice with spot-based clustering, using spot deconvolution with ANCHOR (21) to further annotate clusters in the identified main histological areas of the liver with their respective cell-type abundances within each spot (Figure 1I). After quality control, we obtained a total of 4,769 spots from the sham group and 4,912 spots from the I/R group (average of 2,420 spots per specimen). As each Visium ST spot resolution is limited to 55 μm with each group of cells captured, we leveraged our annotated scRNA-seq data to further deconvolute each ST spot with estimated cell-type compositions to increase resolution (Figure 1I and Supplemental Figure 2F). Our cell-type composition deconvolution data revealed that the evenly distributed hepatic immune cells in sham livers were dramatically redistributed after liver I/R. A marked abundance of neutrophils was found in the necrotic area of the I/R liver, illustrated in Figure 1I. This approach revealed that neutrophils became highly enriched in necrotic pericentral zones, while other immune populations were reduced or displaced following I/R.

### Transcriptional landscape of hepatic immune subsets following liver I/R.

To comprehensively characterize how distinct hepatic immune cell populations respond to surgical stress, we performed subset-level transcriptional analyses across adaptive and innate cells in the hepatic immune microenvironments using scRNA-seq and ST data. Within CD3^+^ T cells, total CD4^+^ and CD8^+^ T cell numbers did not significantly change following I/R (Figure 1, C and F). Subset-level analysis of CD4^+^ T cells identified 6 transcriptional subpopulations based on differentially expressed genes (DEGs), including T helper 1 (Th1) effector/memory cell, Th2, regulatory T cell (Treg), IFN-stimulated, and *Hsp^+^* subsets (Supplemental Figure 3, A–C). Th1 memory and Th1 effector subsets were enriched in sham livers, whereas Th2 subsets were more prevalent after I/R, resulting in a decreased Th1/Th2 ratio (Supplemental Figure 3C). Differential expression analysis revealed increased *Txnip*, *S100a9*, *Ctla2a*, *Ifngr1*, *Ccl5*, and *Rgs1* and reduced *Cd52* expression in I/R–associated CD4^+^ T cells (Supplemental Figure 3D). Both Gene Ontology biological process (GO-BP) and Kyoto Encyclopedia of Genes and Genomes (KEGG) pathway analysis indicated modest enrichment of T cell differentiation and IL-17 signaling pathways, alongside suppression of activation-associated processes (Supplemental Figure 3D). ST analysis showed weak and evenly distributed CD4^+^ cells across lobular zones in both conditions (Supplemental Figure 3E). CD8^+^ T cells formed 3 transcriptional subclusters without significant changes in overall abundance after I/R (Supplemental Figure 4A). Within the NKT compartment, 3 subtypes were identified; both type 1 and type 2 NKT cells (22) decreased after I/R, whereas an S100a8/a9^+^ subpopulation increased (Supplemental Figure 4B). Both CD8^+^ T cells and NKT cells exhibited upregulation of *S100a8* and *S100a9* in I/R livers (Supplemental Figure 4, C and D), and KEGG analysis demonstrated enrichment of IL-17 signaling pathways among upregulated genes (Supplemental Figure 4, E and G). ST mapping revealed limited redistribution of CD8^+^ T cells and NKT cells, with only sparse localization near pericentral regions following I/R (Supplemental Figure 4, F and H). Together, these findings indicate that while CD3^+^ T cell abundance remains largely stable during early reperfusion, subset-level transcriptional changes occur without marked spatial reorganization.

Total B-cell abundance was altered, with scRNA-seq revealing changes in the composition of specific B-cell subsets. We characterized 8 distinct subpopulations of B cells among 11 subclusters — naive, memory, follicular, activated, B1, antibody-secreting cells/plasma cells, transitional, and antigen-presenting B cells — based on their canonical feature genes (23). Memory and follicular B cells were enriched in sham livers, whereas activated B cells predominated after I/R (Supplemental Figure 5, A–C). I/R–associated activated B cells upregulated *Cd83*, *Junb*, *Satb1*, and *Cxcr4* and showed reduced expression of *Cd79a* and *Cd79b*, which encode 2 subunits (Igα and Igβ) of the B cell receptor (BCR) complex (24), suggesting impairment of B cell memory development (Supplemental Figure 5D). KEGG pathway analysis indicated decreased BCR signaling but enhanced TNF and IL-17 signaling, along with enrichment of light zone–associated gene signatures (Supplemental Figure 5E). ST analysis demonstrated sparse periportal localization in sham livers and comparable periportal and mid-lobular distribution after I/R, without enrichment in necrotic regions (Supplemental Figure 5F).

scRNA-seq identified 3 NK subsets (NK1–NK3) across 6 subclusters, in line with established feature markers (25) (Supplemental Figure 6, A–C). NK3 cells were predominantly derived from sham livers, whereas NK1 and NK2 subsets were enriched following I/R, indicating altered NK composition (Supplemental Figure 6C). I/R–associated NK1 and NK2 cells upregulated *Il18rap*, *Tnfaip3*, *Ptprc*, and *Il7r*, with GO analysis showing enrichment of lymphocyte differentiation and cytokine-mediated signaling pathways (Supplemental Figure 6D). ST analysis revealed comparable lobular distribution between sham and I/R conditions without enrichment in necrotic regions (Supplemental Figure 6E).

Conventional dendritic cells (cDCs) were reduced following I/R and segregated into 7 transcriptional subsets (26, 27): cDC1, cDC2A, cDC2B, cDC3, inflammatory cDC2, Ccr7^+^, and Ifit^+^ dendritic cells (Supplemental Figure 7, A–C). Inflammatory cDC2, cDC2B, and cDC3 populations were enriched in I/R livers and upregulated *Tgfbi*, *Nfkbia*, *Ccl6*, *Cxcl2*, *Ccr1*, and *Ccr2* (Supplemental Figure 7D). Plasmacytoid dendritic cells (pDCs) were also reduced and distributed across 5 subclusters, with I/R–associated cells enriched in *Il7r^+^* progenitor-derived populations and showing increased *Cd2ap* expression (Supplemental Figure 7, E and F). GO pathway analysis demonstrated enrichment of leukocyte chemotaxis and inflammatory signaling, alongside suppression of antigen presentation pathways with concurrent TNF and IL-17 pathway enrichment (Supplemental Figure 7G). ST analysis showed broad dendritic cell distribution in sham livers but relative exclusion from necrotic regions after I/R (Supplemental Figure 7H).

scRNA-seq revealed marked infiltration of monocytes following I/R, consistent with flow cytometry findings (Supplemental Figure 8A). Classical, non-classical, and intermediate monocyte subsets were identified, along with a dendritic cell–producing monocyte (DCMo) (28) population characterized by *H2-Aa*, *H2-Ab1*, *H2-Eb1*, and *Cd74* expression, which was expanded in I/R livers (Supplemental Figure 8, A–C). I/R–associated monocytes upregulated *Il1b*, *Ccl3*, *Cxcl2*, *Ccr1*, and *Ccr2*, with reduced *Cd74* and *Cxcr4* expression (Supplemental Figure 8D). Infiltrating macrophages (*Adgre1^hi^Clec4f^lo^*) (8) were less abundant with cluster 2 enriched in I/R livers (Supplemental Figure 8E), displaying similar inflammatory signatures with upregulation of *Cd14*, *Tnfaip3*, *Ccl4*, *Ccl9*, *Cxcl2*, and *Cxcl10* (Supplemental Figure 8F). GO-BP pathway analyses for infiltrating monocytes and macrophage populations indicated enrichment of myeloid cell differentiation/migration and innate immune activation pathways, respectively, accompanied by suppression of antigen presentation programs in both cell types (Supplemental Figure 8, G and H). ST analysis demonstrated broad distribution of infiltrating monocytes and macrophages after I/R without preferential accumulation in necrotic regions (Supplemental Figure 8, I and J). A small population of basophils (*n* = 31, *Fcer1a*, *Mcpt8*, *Cd9*) (29) was also detected following I/R, indicating broader innate immune activation, although their relative abundance remained limited (Supplemental Figure 8C). Collectively, integrated single-cell and spatial analyses demonstrate that liver I/R induces coordinated but subset-specific transcriptional changes across adaptive and innate immune compartments.

### KCs exhibit inflammatory activation and metabolic suppression following liver I/R.

**Figure 2:**
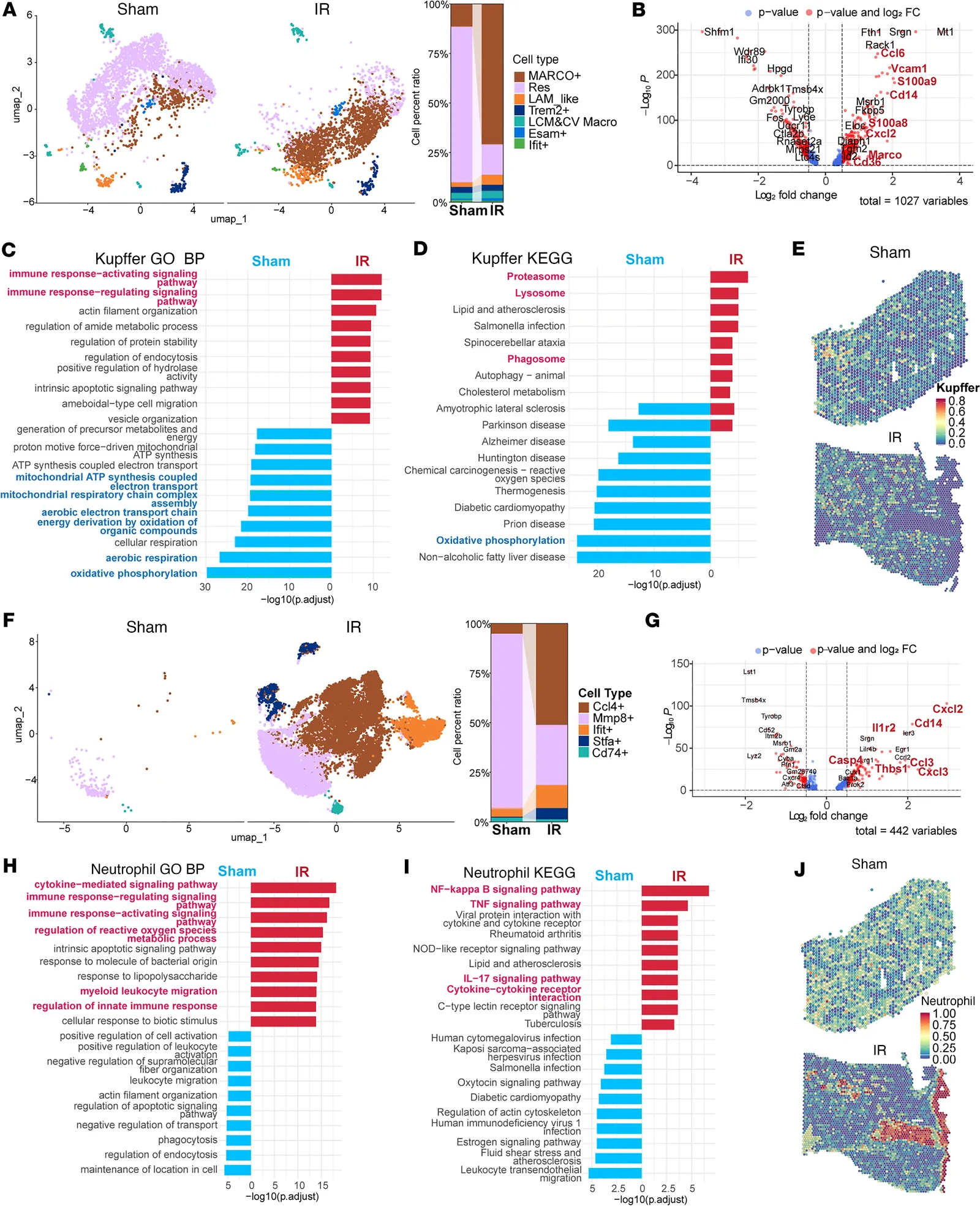
Landscape of KCs and neutrophils in sham and I/R liver. (**A**) UMAP plots show distinct KC subtypes in sham and I/R liver. (**B**) Volcano plot showing DEGs in I/R–associated KCs compared with sham. (**C**) GO biological process pathway enrichment analysis of DEGs in I/R–associated KCs compared with sham. (**D**) KEGG pathway enrichment analysis of DEGs in I/R–associated KCs compared with sham. (**E**) Spatial transcriptomics analysis of KCs in sham and I/R mouse livers. (**F**) UMAP plots showing distinct neutrophil subtypes in sham and I/R liver. (**G**) Volcano plot showing DEGs in I/R–associated neutrophils compared with sham. (**H**) Biological process pathway enrichment analysis of DEGs in I/R–associated neutrophils compared with sham. (**I**) KEGG pathway enrichment analysis of DEGs in I/R–associated neutrophils compared with sham. (**J**) Spatial transcriptomics analysis of neutrophils in sham and I/R mouse livers.

Resident KCs exhibited substantial transcriptional heterogeneity after liver I/R (Supplemental Figure 9A). Based on the DEGs of each subset (Supplemental Figure 9B) and established KCs subtypes (30), we characterized 8 subtypes of KCs from a total of 14 subclusters — KC2 (31) (*Esam^+^*), MARCO^+^ (*Marco^+^*; macrophage receptor with collagenous structure), resident (Res), lipid-associated macrophage–like (LAM-like), liver capsule macrophage (LCM) and central vein (CV) *Marco*^+^, *Trem2^+^*, and Ifit^+^ — in the liver (Figure 2A). Most of the resident KCs were enriched in sham livers, whereas MARCO^+^ subsets predominated in I/R livers (Figure 2A and Supplemental Figure 9C). I/R–associated MARCO^+^ KCs were enriched in gene expression related to proinflammatory responses, adhesion, and infiltration (*Marco*, *Cd14*, *Cd36*, *Vcam1*, *S100a8*, *S100a9*, *Ccl6*, and *Cxcl2*) (Figure 2B). GO-BP analysis revealed that I/R–associated MARCO^+^ KCs were enriched in immune response–activating/regulating signaling pathways but showed suppressed mitochondrial ATP synthesis, aerobic respiration, and oxidative phosphorylation (OXPHOS) pathways, indicating that liver I/R promoted metabolic reprogramming in KCs (Figure 2C). KEGG pathway enrichment analysis showed enrichment in proteasome, lysosome, and phagosome signaling pathways (Figure 2D). Notably, both GO-BP and KEGG analysis revealed a strong suppression of OXPHOS signal pathway in KCs after liver I/R. ST analysis revealed that KCs were broadly distributed across the sham liver tissue and absent from necrotic zones of I/R livers, despite their overall transcriptional activation (Figure 2E). Together, these data indicate that liver I/R induces marked transcriptional shifts within resident KCs, characterized by enhanced inflammatory signaling and coordinated suppression of mitochondrial oxidative metabolism.

### Neutrophils accumulate in necrotic areas and exhibit inflammatory activation after liver I/R.

Neutrophils represented the most markedly expanded myeloid compartment following liver I/R. Similarly to infiltrating monocytes, nearly all captured neutrophils originated from I/R mice, forming 5 transcriptionally distinct clusters (Supplemental Figure 9D). Subset analysis identified *Ccl4^+^*, *Mmp8^+^*, *Ifit^+^*, *Stfa^+^*, and *Cd74^+^* neutrophils, consistent with previously reported inflammatory phenotypes (18) (Figure 2F and Supplemental Figure 9). Notably, the *Stfa^+^* subset (cluster 3) was characterized by high expression of *Stefin A* family genes (*Stfa1*, *Stfa2*, *Stfa2l1*, *BC100530*), a signature associated with bone marrow–derived neutrophil expansion in severe infection models, including SARS-CoV-2 (32, 33) (Supplemental Figure 9E). Neutrophils in I/R livers upregulated numerous inflammatory and danger-response genes (*Cd14*, *Casp4*, *Thbs1*, *Il1r2*, *Ccl3*, *Cxcl2*, *Cxcl3*) (Figure 2G). GO-BP enrichment analysis highlighted cytokine signaling, ROS metabolism, innate immune activation, and leukocyte migration (Figure 2H). KEGG analysis showed increased activation of NF-κB, TNF, IL-17, and cytokine–cytokine receptor interaction pathways, further supporting a robust proinflammatory phenotype (Figure 2I). These transcriptional programs underscore the central role of infiltrating neutrophils as key effectors of sterile liver inflammation during I/R. ST analysis revealed a distinct localization pattern for neutrophils compared with other immune cells. In I/R livers, neutrophils were uniquely localized and highly enriched within necrotic areas, in contrast to KCs and monocytes, which were largely excluded from these zones (Figure 2J). This spatial segregation suggests direct engagement of neutrophils with injured parenchyma and positions them uniquely within the inflammatory niche generated after reperfusion.

### Neutrophil-KC interaction is selectively enriched via TSP-1/CD36 signaling after liver I/R.

**Figure 3:**
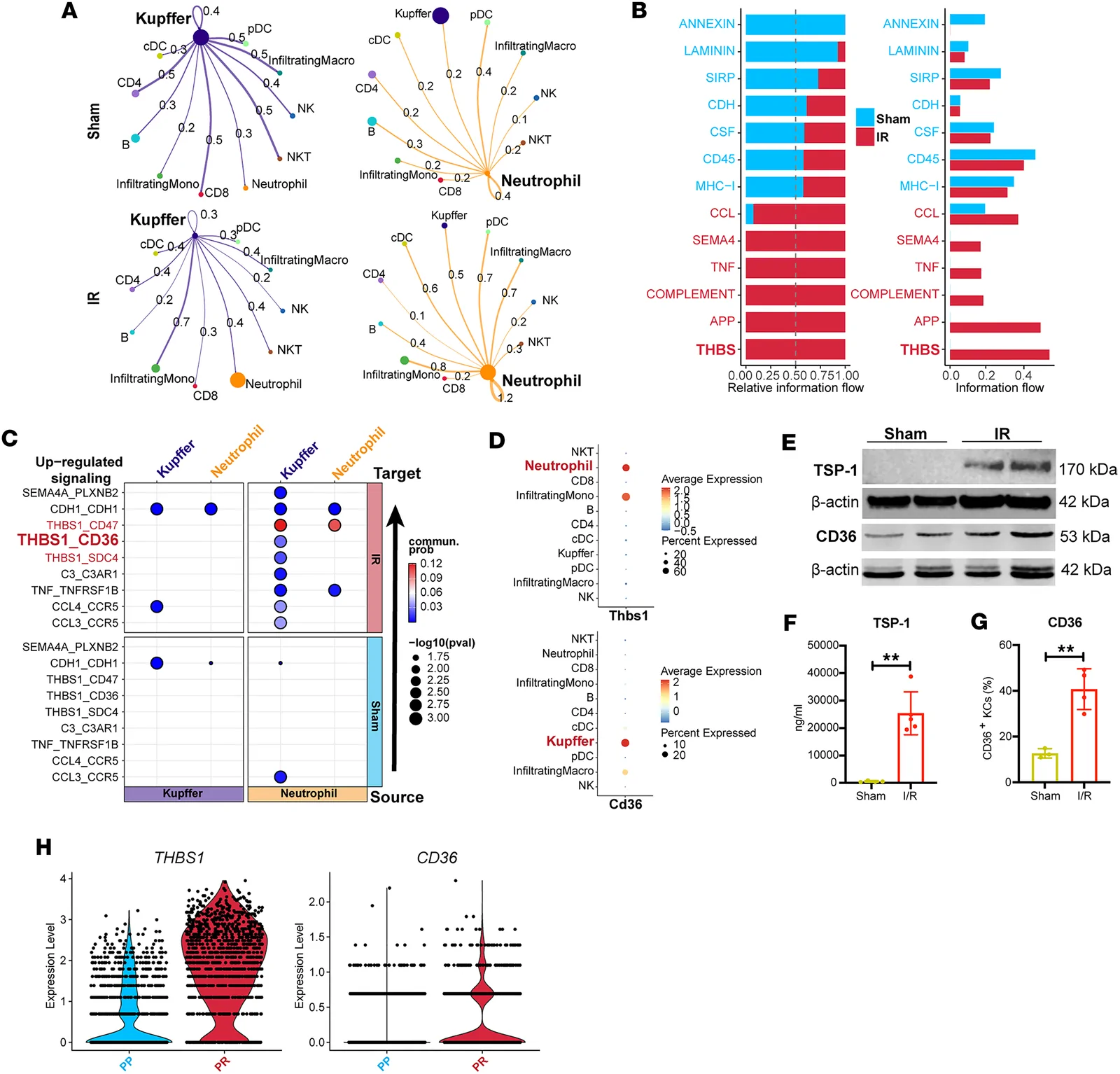
Neutrophil-KC interaction is selectively enriched via TSP-1/CD36 signaling after liver I/R. (**A**) Circle plot showing predicted outgoing cell-cell interactions from KCs and neutrophils to other immune cell subsets in sham (top) and I/R (bottom) liver using CellChat analysis. (**B**) Bar graph showing the relative information flow of ligand-receptor interactions in neutrophil-to-KC communication in sham and I/R liver. (**C**) Bubble plot showing upregulated source-to-target signaling pathways from KCs (left) or neutrophils (right) in sham (bottom) and I/R (top) liver. (**D**) Dot plot showing *Thbs1* and *Cd36* expression in hepatic immune cells. (**E**) Western blot showing TSP-1 and CD36 expression in sham and I/R liver. (**F**) ELISA showing TSP-1 serum levels from sham and I/R mice. (**G**) Flow cytometry showing frequencies of CD36^+^ KCs from sham and I/R liver tissues. (**H**) Violin plot showing THBS1 expression in neutrophils and CD36 expression in KCs in post-reperfusion (PR) patient samples compared with pre-procurement (PP) ones. Data represent the mean ± SEM. **P < 0.01, by 2-tailed, unpaired *t* test (**F** and **G**).

To determine whether liver I/R alters intercellular crosstalk within the hepatic immune microenvironment, we performed cell-cell interaction analysis using CellChat (34). Both global communication strength and number of predicted interactions were markedly increased following I/R injury compared with sham (Supplemental Figure 10A). Ligand-receptor analysis revealed that among all enriched pathways, thrombospondin (THBS) signaling emerged as the most strongly upregulated intercellular signaling axis after I/R and was predominantly initiated from neutrophils and infiltrating monocytes to other immune cells in the liver (Supplemental Figure 10 , B and C). Given that KCs represent the largest immune population under homeostasis and neutrophils become predominant after I/R (Figure 1, E and F), we focused on these two cell types. Notably, the interactions of KCs with most other immune cell types decreased after I/R, besides infiltrating monocytes and neutrophils. I/R markedly augmented the interactions of neutrophils with other cell types, but not with CD4^+^ and CD8^+^ T cells (Figure 3A and Supplemental Figure 10D). THBS signaling emerged as the most enriched neutrophil-derived signaling pathway to all other immune cells following I/R (Supplemental Figure 10E) and was also the top upregulated signaling pathway from neutrophils to KCs (Figure 3B). Specifically, among thrombospondin-1 (TSP-1) interactions with CD47, CD36, and SDC4, TSP-1/CD36 signaling was selectively and uniquely augmented in neutrophil-to-KC interactions (Figure 3C).

Consistent with these predictions, *Thbs1*, which encodes TSP-1, was predominantly expressed in both infiltrating monocytes and neutrophils (Figure 3D) and also dramatically increased in all subtypes of neutrophils following liver I/R (Supplemental Figure 10F). In parallel, *Cd36*, a scavenger receptor involved in lipid metabolism and cellular signaling (35) and a receptor of TSP-1 (36), exhibited the highest expression among hepatic immune populations within KCs (Figure 3D) and was significantly enriched in I/R–associated KC subsets (Supplemental Figure 10F). Transcriptomic findings were supported by significantly increased serum levels of TSP-1 (Figure 3E) and enhanced protein expression in the infiltrated neutrophils obtained from ischemic liver (Figure 3F). Consistently, elevated frequencies of CD36^+^ KCs were observed by flow cytometry after liver I/R (Figure 3G and Supplemental Figure 10G).

To assess translational relevance, we analyzed a publicly available scRNA-seq dataset (GSE171539) obtained from human liver transplantation (37) and evaluated the interaction between neutrophils and KCs before and after organ reperfusion (Supplemental Figure 11, A–C). Expression of *THBS1* in neutrophils and *CD36* in KCs was significantly upregulated in post-reperfusion (PR) samples compared with pre-procurement ones (Figure 3H). Collectively, these transcriptional, protein-level, and cross-species data identify neutrophil-derived TSP-1 and KC-expressed CD36 as a selectively enriched signaling axis during liver I/R, suggesting a mechanism through which infiltrating neutrophils may modulate KC functional states within the injured hepatic microenvironment.

### Spatial transcriptomics reveals enhanced neutrophil-KC interaction after liver I/R.

**Figure 4:**
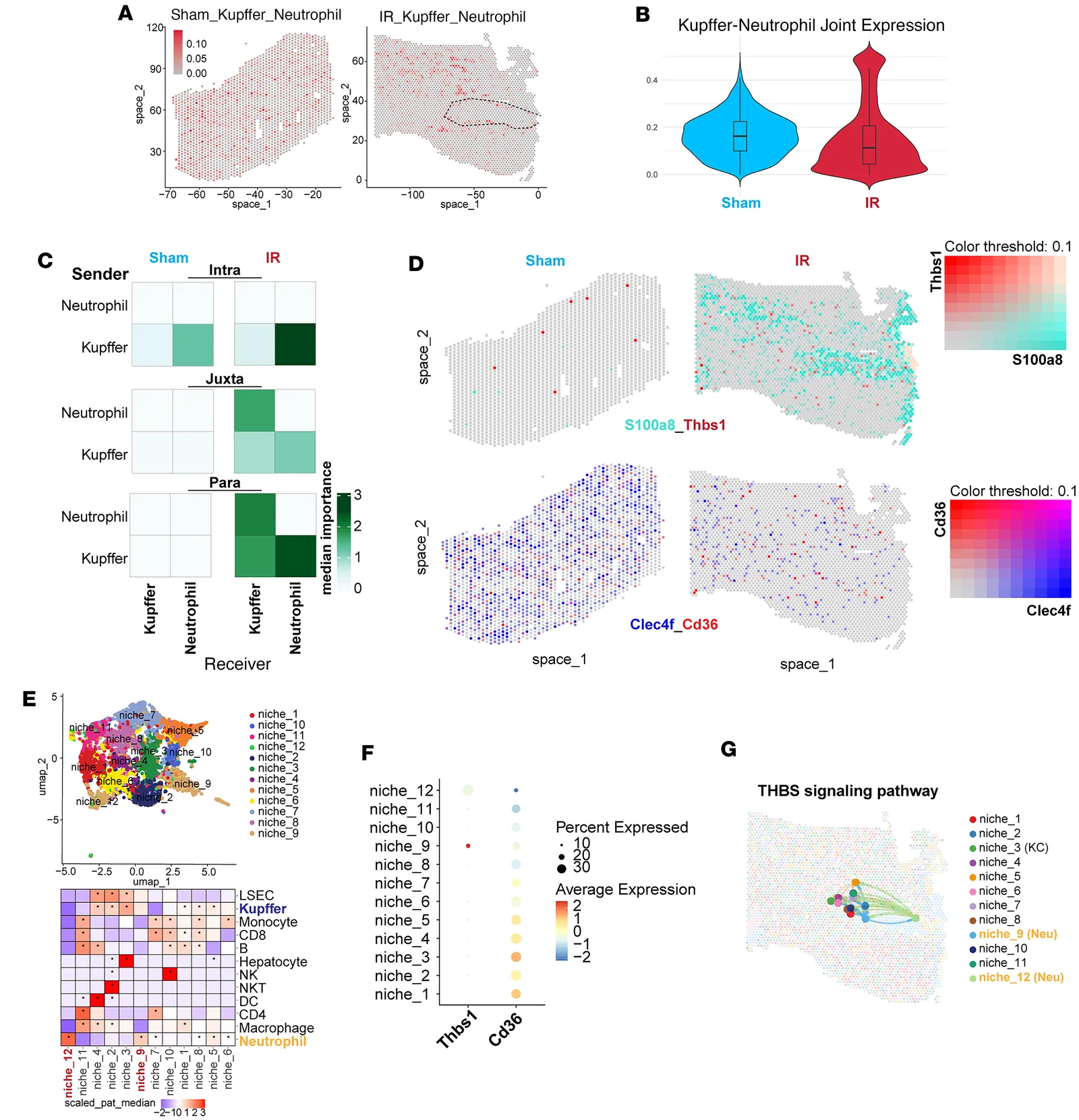
Spatial transcriptomics reveals enhanced neutrophil-KC interaction after liver I/R. (**A**) Spatial distribution of niches defined by joint KC and neutrophil marker expression in sham (left) and I/R (right) liver tissues. (**B**) Violin plot showing KC-neutrophil joint expression. (**C**) Heatmap analysis using MISTy to evaluate spatial dependencies between KCs and neutrophils within 3 neighborhood contexts: colocalization within the same spot (Intra), immediate neighborhood (Juxta), and extended neighborhood (Para). (**D**) Spatial colocalization of *Thbs1* with neutrophils (*S100a8*) and *Cd36* with KCs (*Clec4f*) in sham and I/R liver. (**E**) UMAP visualization of spatial transcriptomics data identifying cell-type niches in the hepatic microenvironment. (**F**) Dot plot showing the spatial distribution of *Thbs1* and *Cd36* in the liver. (**G**) Spatial visualization of THBS signaling pathway by CellChat analysis.

To determine whether the predicted neutrophil-KC interaction was also reflected in the hepatic spatial architecture, we interrogated our ST data by evaluating joint expression of KCs and neutrophils. In sham livers, neutrophils and KCs exhibited relatively dispersed and evenly distributed colocalization (Figure 4A). Notably, distribution of the joint expression scores of KCs and neutrophils in the liver of sham and I/R mice revealed a marked upward shift in the expression signature (score > 0.4) in the I/R group compared with the sham control, indicating an enhanced immune cell crosstalk after I/R (Figure 4B). To quantify the spatial dependency, we applied MISTy (38) analysis, defining 3 levels of spatial proximity: intra-spot (colocalization), juxta (immediate neighborhood, radius = 1 spot), and para (extended neighborhood, radius = 5 spots). Broader spatial analysis using MISTy revealed enhanced immune cell spatial dependencies, particularly in the juxta and para regions, after I/R (Supplemental Figure 12A). I/R markedly increased spatial codependency between KCs and neutrophils across all spatial scales in comparison with sham controls, in alignment with our CellChat results (Figure 4C). Spatial colocalization analysis further demonstrated overlap of *Thbs1* with the neutrophil marker *S100a8* and of *Cd36* with the KC marker *Clec4f* in the I/R livers, supporting activation of the TSP-1/CD36 signaling axis in situ (Figure 4D).

To further dissect the spatial architecture of hepatic immune microenvironments during I/R injury, we extended our analysis to characterize spatial-specific distribution patterns of major hepatic cell types. Leveraging an established approach (38) to clustering all ST spots based on cell-type composition, we identified 12 distinct cell-type niches (Figure 4E). Integration with scRNA-seq annotations revealed 7 leukocyte-rich niches (1, 2, 4, 6, 7, 10, and 11; composed of macrophages, monocytes, dendritic cells, NK cells, B cells, and other lymphoid populations), 2 myeloid niches (5 and 8), 2 neutrophil-specific niches (9 and 12), and 1 parenchymal niche (3; enriched in hepatocytes and liver sinusoidal endothelial cells). In sham livers, the parenchymal niche was diffusely distributed; however, following I/R, neutrophil-rich niches became spatially restricted to necrotic zones (Figure 4E and Supplemental Figure 12B), indicating the spatial compartmentalization of inflammatory responses. Consistent with transcriptional findings, *Thbs1* expression was selectively enriched within neutrophil-dominant niches, whereas *Cd36* was preferentially enriched in KC-rich niches (Figure 4F). Notably, ST mapping of I/R liver further revealed that THBS signaling originated from necrotic zones and radiated outward toward adjacent immune-enriched areas (Figure 4G and Supplemental Figure 12C).

### Metabolic reprogramming of KCs after I/R associates with CD36-enriched signaling.

**Figure 5:**
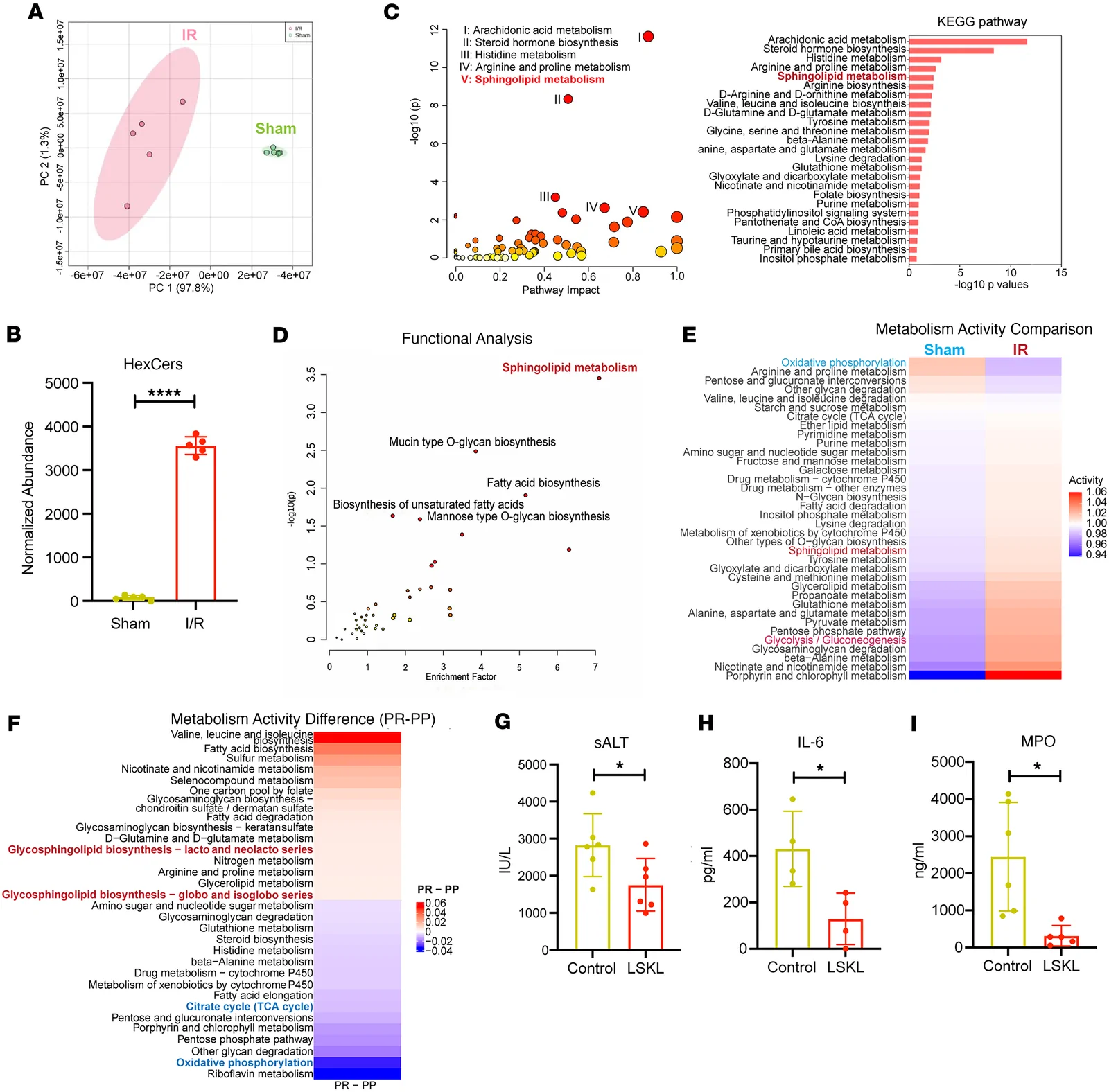
Metabolic reprogramming of KCs after I/R associates with CD36-enriched signaling. (**A**) Principal component analysis (PCA) of non-targeted metabolomics profiling in I/R-KCs compared with sham-KCs. (**B**) Normalized hexosylceramide (HexCer) levels in sham and I/R KCs. (**C**) KEGG enrichment analysis of altered metabolic profiles in I/R-KCs compared with sham-KCs. (**D**) Scatterplot showing the top enriched functional metabolic pathways in I/R-KCs compared with sham-KCs based on metabolomics data. (**E**) Heatmap comparing metabolic pathway activity between sham-associated KCs and I/R–associated KCs based on scRNA-seq data. (**F**) Heatmap comparing metabolic pathway activity in KCs based on human scRNA-seq data from liver transplant patients (GSE171539). (**G**) Serum alanine aminotransferase (sALT) levels were quantified in I/R mice treated with control or LSKL peptide. (**H**) Serum IL-6 levels were quantified in I/R mice treated with control or LSKL peptide. (**I**) Serum myeloperoxidase (MPO) levels were quantified in I/R mice treated with control or LSKL peptide. Data represent the mean ± SEM. *P < 0.05, ****P < 0.0001, by 2-tailed, unpaired *t* test (**B** and **G**–**I**).

We have recently shown that metabolic reprogramming of KCs plays a central role in shaping immune responses during hepatic I/R to exacerbate liver damage (8). Given the enrichment of CD36 expression in I/R–associated KCs and its known role in lipid uptake (39), we next investigated whether liver I/R metabolically reprograms KCs and whether this reprogramming is associated with TSP-1/CD36. Untargeted steady-state metabolomics profiling (40) of sorted KCs revealed a distinct metabolic signature in I/R samples compared with sham controls, as demonstrated by principal component analysis (PCA) (Figure 5A).

It has been shown that ceramides, as precursors of predominant complex sphingolipids, play a key role in cell stress responses during I/R injury (41). Lipidomic analysis identified marked accumulation of hexosylceramides, including glucosylceramides and galactosylceramides, in I/R–associated KCs (Figure 5B). Given that CD36 promotes the ceramide-to-glucosylceramide conversion pathway (42), this shift strongly implicates CD36 activity in ceramide flux during I/R. Consistently, both KEGG enrichment analysis and overall global pathway analysis highlighted enrichment of sphingolipid metabolism as markedly upregulated and the most enriched metabolic change pathway in I/R-KCs (Figure 5, C and D), suggesting that TSP-1/CD36 signaling contributes to the regulation of sphingolipid homeostasis. To complement metabolomic findings, we performed a complementary metabolic pathway scoring approach (43) using our scRNA-seq data. I/R–associated KCs demonstrated enrichment of glycolysis and CD36-associated lipid metabolic programs, including sphingolipid metabolism, fatty acid degradation, and glycerolipid metabolism. In parallel, pathways related to the tricarboxylic acid (TCA) cycle and OXPHOS were suppressed, consistent with earlier transcriptional analyses (Figure 5E).

To assess the translational relevance of these findings, we used scRNA-seq dataset GSE171539 (37) again to analyze metabolic pathway activity in human KCs during liver transplantation. Consistent with our murine data, PR-KCs exhibited enhanced glycosphingolipid biosynthesis activity, including globo-, lacto-, and neolacto-series pathways (Figure 5F), which is strongly associated with CD36 function and ceramide processing and supports the role of TSP-1/CD36 signaling in driving immune-metabolic reprogramming of KCs across species. Notably, TCA cycle and OXPHOS were found to be suppressed in PR-KCs, consistent with enriched glycolysis in our metabolic analysis of mouse scRNA-seq data. This metabolic profile mirrors that of classically activated macrophages when engaging in potent inflammatory responses (44). Collectively, these results demonstrate that liver I/R is associated with extensive immune-metabolic reprogramming in KCs, including enhanced sphingolipid biosynthesis and suppressed oxidative metabolism. These findings support a model in which CD36-enriched signaling may contribute to the metabolic state of KCs during hepatic I/R injury.

### Pharmacologic inhibition of TSP-1 attenuates liver injury after I/R.

Previous studies have implicated TSP-1/CD36 signaling in mediating tissue damage during kidney I/R injury (36). To directly evaluate the role of TSP-1 during hepatic I/R in vivo, we treated I/R–subjected mice with TSP-1 inhibitor (LSKL peptide, sulfosuccinimidyl oleate) (45, 46). Pharmacologic blockade of TSP-1 substantially protected the liver from I/R injury, as evidenced by decreased serum level of alanine aminotransferase activity in comparison with vehicle-treated controls (Figure 5G). In parallel, circulating IL-6 and myeloperoxidase were also markedly decreased in LSKL-treated mice compared with controls, suggesting attenuation of I/R–induced inflammation and neutrophil-associated oxidative stress (Figure 5, H and I). These findings provide in vivo support for the idea that TSP-1 contributes to the severity of liver injury following surgical stress. In the context of our transcriptional, spatial, and metabolomic analyses demonstrating increased neutrophil-derived TSP-1 and elevated CD36 expression in KCs, pharmacologic inhibition of TSP-1 is consistent with disruption of this signaling axis during I/R. While additional studies are required to define cell type–specific mechanisms, these data indicate that TSP-1 signaling represents a promising therapeutic strategy to mitigate the inflammatory consequences of hepatic I/R.

## Discussion

Surgical stress, such as hepatic I/R injury, triggers a robust immune response in the liver, leading to local and systemic inflammation and the activation of diverse immune cell populations (3). Beyond its metabolic and detoxifying function, the liver functions as a crucial immunological organ enriched with resident and infiltrating immune subsets that collectively shape postoperative outcomes (47, 48). In this study, we applied an integrated single-cell multiomics approach, comprising scRNA-seq, spatial transcriptomics, high-dimensional spectral flow cytometry, and unbiased metabolomics, to comprehensively map the hepatic immune landscape and dissect the cellular and molecular mechanisms at spatial, transcriptomic, and protein levels underlying immune responses to surgical stress. Our study not only establishes a high-resolution immune atlas of the liver. Specifically, we reveal spatially distinct immune zonation, altered immune cell phenotypes, and cell-cell interaction networks driven by liver I/R injury.

Single-cell multiomics has emerged as a powerful tool to delineate the genome, epigenome, transcriptome, proteome, and metabolome of complex tissues at the single-cell level by simultaneously integrating various single-modality omics methods (49, 50). These approaches have identified cholangiocyte and mesenchymal heterogeneity in the human liver (51) and provided a map of the human hepatic immune microenvironment (52). More recently, a combination of single-cell multiomics, spatial omics, massively parallel reporter assays, and a hierarchical deep learning model have been leveraged to successfully map enhancer-gene regulatory networks across mouse liver cell types, together with other core hepatocyte transcription factors (53). Further, using zone-specific single-cell transcriptomics, spatial transcriptomics, and intravital imaging, a subset of immunosuppressive macrophages enriched in periportal vein zones that express high levels of IL-10 and MARCO has been identified as being decreased in chronic liver inflammatory diseases such as primary sclerosing cholangitis (PSC) and metabolic dysfunction–associated steatohepatitis (MASH) (10). Additional studies have employed multiomics modalities, including scRNA-seq, single-nucleus RNA sequencing, and spatial transcriptomics, to generate a comprehensive atlas of the PSC liver (54) and to define a hepatocyte-cholangiocyte plasticity and zonation loss in MASH patients (55). However, an integrated and spatially resolved immune-metabolic characterization of hepatic I/R injury has been lacking. By combining transcriptional, spatial, and metabolic profiling across murine and human datasets, our study provides a cross-species resource to interrogate immune cell interactions during surgical stress.

Building on this foundation, our study highlights a remarkable immune cell number, immune zonation, and phenotypic changes in the hepatic microenvironment and demonstrates the critical interaction between neutrophils and KCs in liver injury, echoing the complexities of resident and infiltrating immune cell interactions within the liver immune microenvironment (56, 57). Although hepatic zonation is well characterized (58, 59), the compartmentalization of immune cells in the liver remains poorly defined. While a recent study used spatial transcriptomics to characterize the zonation of hepatic I/R injury in mice and identified the pericentral zone as most vulnerable based on histological and transcriptional alterations (60), our study advances this understanding by integrating single-cell multiomics to comprehensively map both resident and infiltrating immune populations across injury zones. Importantly, we uncover conserved immune-metabolic crosstalk between KCs and infiltrating neutrophils mediated by TSP-1/CD36 signaling, a mechanistic insight not addressed in previous zonation-focused studies.

Among hepatic immune cells, KCs have emerged as key mediators of I/R–induced inflammation. Consistent with the prior findings that depletion of KCs confers protection against liver I/R (61), our data show that KCs undergo subset redistribution and increased expression of inflammatory mediators after I/R. Importantly, transcriptional analysis reveals suppression of oxidative phosphorylation (OXPHOS) and enrichment of glycolytic and lipid metabolic pathways, indicating altered energetic states during early reperfusion. These findings align with prior work demonstrating that metabolic state influences macrophage functional polarization. While our previous studies have highlighted protective, antiinflammatory reprogramming of KCs (8), our current data indicate that acute surgical stress is associated with a distinct metabolic configuration characterized by reduced mitochondrial respiration and enhanced sphingolipid-associated programs.

A central finding of this study is the selective enrichment of TSP-1 signaling in neutrophil-KC interaction following I/R. *Thbs1* expression was markedly induced in infiltrating neutrophils, whereas *Cd36* was enriched in I/R–associated KCs. Pharmacologic inhibition of TSP-1 attenuated liver injury and inflammatory markers, providing functional support for the involvement of this pathway during surgical stress. CD36 facilitates the uptake of long-chain fatty acids (39), driving metabolic reprogramming in macrophages by shifting their energy production from OXPHOS toward fatty acid oxidation and glycolysis in the tumor microenvironment (62). In the liver, CD36^+^ KCs play a regulatory role in oxidative stress, particularly in obesity-related conditions. Silencing CD36 has been shown to reduce levels of malondialdehyde and ROS, indicating its central role in lipid peroxidation and inflammation (63). In our model, the accumulation of hexosylceramides and enriched glycosphingolipid biosynthesis pathways in KCs are consistent with altered ceramide flux in the setting of increased CD36 expression. While our data support an association between neutrophil-derived TSP-1, CD36 enrichment, and metabolic alterations in KCs, further studies using cell type–specific perturbations will be required to establish direct mechanistic causality.

In parallel, a recent study by Weng et al. reported that TSP-1 drives CD8^+^ T cell exhaustion in cancer through CD47-dependent signaling, identifying a chronic, tumor-associated pathway that shapes adaptive immune dysfunction (64). In contrast, our multiomics analysis reveals an acute role of TSP-1 in hepatic sterile inflammation via CD36, highlighting the diverse receptor usage and downstream immune consequences of TSP-1. Determining how TSP-1 receptor specificity (CD36 versus CD47) is modulated across tissues and injury states, and whether selective specific targeting of these pathways can mitigate surgical injury without impairing antitumor immunity, represents an important future direction.

Importantly, our study includes single-cell transcriptomic data from human liver samples subjected to I/R, representing the only publicly available human dataset capturing hepatic immune cells (37). Integration of publicly available human liver transplantation scRNA-seq data revealed increased *THBS1* expression in neutrophils and elevated *CD36* expression in KCs after reperfusion, accompanied by parallel metabolic pathway alterations. These cross-species findings reinforce the relevance of the neutrophil-KC axis during surgical stress and support translational significance.

Overall, our integrative multiomics analyses define a spatially organized immune-metabolic circuit in which infiltrating neutrophils and resident KCs interact within necrotic zones during hepatic I/R. The selective enrichment of TSP-1/CD36 signaling and associated alterations in KC lipid metabolism provide insight into how intercellular communication may influence inflammatory outcomes following surgical stress.

### Limitations of the study.

The cohort size is modest, and spatial transcriptomic profiling was performed on limited samples, which may restrict assessment of inter-individual variability. Although pharmacologic inhibition of TSP-1 attenuated liver injury, cell type–specific genetic approaches will be necessary to definitively define the contribution of neutrophil-derived TSP-1 and CD36-expressing KCs. In addition, while transcriptional and metabolomic data indicate coordinated metabolic alterations, direct functional metabolic flux measurements in vivo were not performed. Future studies incorporating conditional deletion models and metabolic tracing will be important to refine these mechanisms.

## Methods

### Sex as a biological variable

Our study exclusively examined male mice since estrogen influences the immune response to liver I/R process (65). Published human data are from both male and female participants.

### Animals

Male wild-type (C57BL/6) mice (8–12 weeks old) were purchased from The Jackson Laboratory.

### Liver I/R

The surgical procedure has been described in detail (66). Briefly, the left and median liver lobes were occluded with a microvascular clamp (Fine Science Tools) for 60 minutes, and reperfusion was initiated by removal of the clamp; the total reperfusion time was 6 hours. In studies involving TSP-1 inhibition, mice received an intraperitoneal injection of 30 mg/kg per mouse TSP-1 inhibitor LSKL peptide immediately after ischemia (45). Sham animals underwent anesthesia, laparotomy, and exposure of the hepatic hilum without hepatic ischemia.

### Liver damage assessment

Serum alanine aminotransferase was measured using the DRI-CHEM 4000 Chemistry Analyzer System (HESKA). Histological evaluation by H&E staining was used for measuring liver and lung injury. Liver samples were harvested and processed for H&E as previously described (67). The tissues were assessed for the amount (percentage) of inflammation (sinusoidal congestion, cytoplasmic vacuolization, infiltrating inflammatory cells) and necrosis for characterizing liver damage.

### High-dimensional spectral flow cytometry

Mouse liver non-parenchymal cells (NPCs) were isolated as previously described (68, 69). Briefly, after laparotomy, the portal vein was cannulated, and the liver was flushed with HBSS (Invitrogen Life Technologies) supplemented with 0.96 g sodium bicarbonate/500 mL (perfusate I). Then, the liver was perfused with 0.2% protease (Sigma-Aldrich) in perfusate I for 3 minutes, after which it was excised, placed in perfusate II, and diced into 2- to 3-mm pieces. NPCs were separated from the hepatocytes by differential centrifugation (50 RCF for 5 minutes). The supernatant was centrifuged further (300 RCF for 5 minutes twice) to obtain NPCs. Isolated cells were incubated with Fc blocking antibody (BD Pharmingen) and a fixable viability dye (Live/Dead Aqua, Thermo Fisher Scientific), followed by surface staining with the following fluorochrome-conjugated antibodies: CD11b-BV750 (BioLegend, catalog 101267, clone M1/70), F4/80-APC (BioLegend, catalog 123116, clone BM8), Gr-1–PerCP (BioLegend, catalog 108426, clone RB6-8C5), Ly6C-BV605 (BioLegend, catalog 128036, clone HK1.4), Ly6G–Alexa Fluor 700 (BioLegend, catalog 127622, clone 1A8), CD11c-BUV563 (BD Biosciences, catalog 749040, clone N418), CLEC4F–Alexa Fluor 647 (BioLegend, catalog 156804, clone 3E3F9), NK1.1–PE/Cy7 (BioLegend, catalog 108714, clone PK136), B220–PE/Cy5 (BioLegend, catalog 126514, clone RA3-6B2), CD3-BUV661 (BD Biosciences, catalog 741562, clone 17A2), CD4-BUV496 (BD Biosciences, catalog 741050, clone RM4-5), CD8a–Pacific blue (BioLegend, catalog 100728, clone 53-6.7), and I-A/I-E–Brilliant Violet 711 (BioLegend, catalog 107643, clone M5/114.15.2). We used the UMAP algorithm implemented in OMIQ software (http://omiq.ai) for data dimensionality reduction. The marker expression across the cell islands was visualized on UMAP plots and indicated cell types present within the cell islands. FlowSOM clustering algorithm was used to distinguish cell populations present within the cell islands and visualized on the UMAP plots. A clustered heatmap analysis implemented in OMIQ was created to give an overview of marker expression within each cluster. Immune cell types were annotated by visual investigation of median marker expression across clusters in heatmaps and expression of these markers in cell islands as visualized on UMAP space.

### Single-cell RNA sequencing

#### Sample preparation.

Samples were prepared as outlined by the 10x Genomics Single Cell 3′ Reagent Kit v3 user guide. Briefly, the samples were washed twice in PBS (Life Technologies) plus 0.04% BSA (Sigma-Aldrich) and resuspended in PBS plus 0.04% BSA. Sample viability was assessed via trypan blue (Thermo Fisher Scientific) and using a hemocytometer (Thermo Fisher Scientific). After counting, the appropriate volume for each sample was calculated for a target capture of 6,000 cells. Samples below the required cell concentration as defined by the user guide (i.e., <400 cells/μL) were pelleted and resuspended in a reduced volume and counted again using a hemocytometer before loading onto the 10x Genomics Single Cell A Chip. After droplet generation, samples were transferred onto a pre-chilled 96-well plate (Eppendorf) and heat-sealed, and reverse transcription was performed using a Veriti 96-well thermal cycler (Thermo Fisher Scientific). After the reverse transcription, cDNA was recovered using Recovery Agent provided by 10x Genomics followed by a Silane DynaBead clean-up (Thermo Fisher Scientific) as outlined in the user guide. Purified cDNA was amplified for 12 cycles before being cleaned up using SPRIselect beads (Beckman). Samples were diluted 4:1 (elution buffer [QIAGEN]/cDNA) and run on a Bioanalyzer (Agilent Technologies) to determine cDNA concentration. cDNA libraries were prepared as outlined by the Single Cell 3′ Reagent Kit v3 user guide with appropriate modifications to the PCR cycles based on the calculated cDNA concentration (as recommended by 10x Genomics).

#### Sequencing.

The molarity of each library was calculated based on library size as measured using a Bioanalyzer (Agilent Technologies) and qPCR amplification data (KAPA, Roche). Samples were pooled and normalized to 10 nM, then diluted to 2 nM using elution buffer (QIAGEN) with 0.1% Tween 20 (Sigma-Aldrich). Each 2 nM pool was denatured using 0.1 N NaOH at equal volumes for 5 minutes at room temperature. Library pools were further diluted to 20 pM using HT-1 (Illumina) before being diluted to a final loading concentration of 14 pM. One hundred fifty microliters from the 14 pM pool was loaded into each well of an 8-well strip tube and loaded onto a cBot (Illumina) for cluster generation. Samples were sequenced on a HiSeq 2500 with the following run parameters: read 1, 26 cycles; read 2, 98 cycles; and index, 8 cycles. A median sequencing depth of 60,000 reads per cell was targeted for each sample.

#### Gene expression analysis.

Preliminary sequencing results (BCL files) were demultiplexed and converted to FASTQ files with Cell Ranger mkfastq (version 3.0). The FASTQ files were aligned to the mm10 reference genome (GRCm38.91). We applied Cell Ranger count/aggr for preliminary data analysis and generated a file that contained a barcode table, a gene table, and a gene expression matrix (70). R (version 4.0.0) and the Seurat R package (version 3.1) were used for gene expression analysis. Cells with 2,500 genes and a mitochondrial gene percentage of >10% were excluded. After data normalization, principal component analysis (PCA) with variable genes was used as the input and identified significant principal components (PCs) based on the jackStraw function. Fifteen PCs were selected for clustering and nonlinear dimension reduction. Cells were clustered by the FindClusters function and the FindAllMarkers function to find differentially expressed genes between each type of cell and group. Differentially expressed genes between 2 clusters were generated by Wilcoxon’s rank-sum test with adjusted *P* value less than 0.05. The DoHeatmap function was used to draw the heatmaps. Gene Ontology (GO) and Kyoto Encyclopedia of Genes and Genomes (KEGG) enrichment analysis was conducted by enrichGO and enrichKEGG function implemented in the R package clusterProfiler (version 3.16.1). Enriched pathways with adjusted *P* value less than 0.05 were visualized by dot plot function.

### Spatial transcriptomics

#### Visium.

In brief, the mouse liver was harvested and dissected to pieces that fit the 10x Visium capture area. Ten-millimeter sections were cut and placed within the capture area of a 10x Visium Spatial Gene expression slide. Single 10x Visium Spatial Gene expression slides were stored in an airtight container at –80°C until further processing. 10x Visium cDNA libraries were generated according to the manufacturer’s instructions. Tissue sections were fixed in chilled methanol. H&E staining was performed to assess tissue morphology and quality. Tissue was then lysed, and reverse transcription was performed followed by second-strand synthesis and cDNA denaturation. cDNA was transferred to a PCR tube, and concentration was determined by qPCR. Spatially barcoded, full-length cDNA was amplified by PCR. Indexed sequencing libraries were generated via end repair, A-tailing, adapter ligation, and sample index PCR. Full-length cDNA and indexed sequencing libraries were analyzed using the Qubit 4 fluorometer (Thermo Fisher Scientific) and Agilent 2100 Bioanalyzer.

Histological examination of the H&E-stained Visium tissue sections was performed blindly, without knowledge of spatial gene expression. Portal tracts were identified as having visible bile ducts and/or bile ductules. Necrosis was identified on the basis of regional, confluent loss of hepatocyte hematoxylin nuclear staining combined with decreased eosin staining of the cytoplasm.

#### Computational analysis: mapping, gene counting, and demultiplexing.

Processing of raw reads was performed using the open-source ST Pipeline (https://github.com/jfnavarro/st_pipeline). In short, quality trimming was performed, and homopolymer stretches longer than 15 bp were removed. The reads were subsequently mapped to the annotated reference genome (GRCm38 v86 and corresponding GENCODE annotation file) using STAR (version 2.7.11b; https://github.com/alexdobin/STAR). After filtering, PCR duplicates were removed, and gene count matrices were generated.

#### Dimensionality reduction and clustering.

Main computational analysis of spatial read-count matrices was performed using the Seurat package in R. First, count matrices and metadata were loaded, translating Ensembl IDs to gene symbols simultaneously. Reads of individual samples were filtered to keep only protein-coding genes and subsequently normalized using the SCTransform function in Seurat. The created objects were then integrated using canonical correlation analysis (CCA) with the MultiCCA function (https://github.com/almaan/ST-mLiver/blob/dcd67a7a0e9a13743bbcc89d3c5cc9fc1201c663/scripts/MultiCCA.R.). Normalization of integrated data was performed, regressing out sample identities using the SCTransform function in Seurat. Thereafter, the CCA vectors were subjected to shared-nearest-neighbor graph-based clustering via the FindNeighbors and FindClusters functions. For modularity optimization, the Louvain algorithm was used, and clustering was performed at a resolution of 0.5 for clustering granularity.

#### Visualization and spatial annotation of clusters.

To visualize the clusters in low-dimensional space and on the spot coordinates under the tissue, nonlinear dimensionality reduction was performed using UMAP with the CCA vectors as input. Visualization and annotation of identified clusters in UMAP space, on spot coordinates as well as superimposed on the H&E images, were performed using the Seurat and STUtility packages.

#### Differential gene expression analysis and expression programs.

Differential gene expression analysis (DGEA) of genes in identified clusters was performed using the function FindAllMarkers from the Seurat package. Following the default option of the method, differentially expressed genes for each cluster were identified using the non-parametric Wilcoxon’s rank-sum test. Initial thresholds were set to a logarithmic fold change of 0.25 to be considered differentially expressed in a cluster and to be present in at least 10% of the spots belonging to the same cluster. Representative markers for each cluster were further selected, by choosing of genes with a positive logarithmic threshold above 0.5 and an adjusted *P* value below 0.05. *P* value adjustments were based on Bonferroni correction using all genes in the dataset.

#### Spatial map of cell dependencies.

The spatial map of cell dependencies integrates transcriptomic profiles with spatial localization to identify critical cellular interactions and functional dependencies within liver tissue slices. This method leverages MISTy’s (Multiview Intercellular SpaTial modeling framework) implementation in mistyR (v1.2.1) to estimate the interactions of cell types in adjacent spatial regions (38). MISTy is a multiview machine learning framework that allows for the decomposition of ST data into components representing local, neighboring, and global effects. By modeling cell-cell interactions across spatial scales, MISTy reveals how specific cell types depend on the presence and activity of neighboring cells for survival, signaling, and function. In liver tissue, this approach highlights dynamic interactions between hepatocytes, immune cells, and vascular endothelial cells, emphasizing how local microenvironments shape cellular behaviors under physiological or pathological conditions. These spatial maps provide insights into intercellular dependencies and identify therapeutic targets by uncovering key cellular hubs and niche-regulated processes.

#### Niche definitions from spatial transcriptomics data.

Niche definitions involve identifying discrete microenvironments within liver tissue using ST data. The approach was recently introduced, building on the methods and concepts that define functional niches by integrating spatial gene expression and cellular composition (38). Similar principles were applied here to analyze liver slices under sham and I/R conditions. Specifically, gene expression patterns combined with spatial coordinates were used to segment the liver tissue into functional niches enriched for distinct signaling pathways, metabolic activities, or immunological interactions. By leveraging high-resolution spatial clustering and transcriptomic signatures, this analysis identified critical microenvironments and revealed their spatial organization. Comparisons between sham and I/R states highlighted niche-specific changes in cellular composition and function, advancing our understanding of how spatially confined microenvironments drive liver responses to injury.

#### Zonation-based DGEA of immune system process and metabolic pathway markers.

To gain insight into DEGs of immune system processes and metabolic pathways between clusters, DGEA between genes associated with immune system processes and metabolic pathways (KEGG) was performed. Each resulting list was cross-referenced with the normalized spatial data, and the expression matrix was subset according to the respective cell type followed by DGEA between clusters with a log fold change threshold of 0.01 and significance (adjusted *P* value) below 0.05.

#### scRNA-seq data.

Our scRNA-seq data were analyzed to compare and complement our ST data. For comparative analysis and visualization, scRNA-seq data were analyzed using the Seurat package (v4.2.0). The count data were first filtered for mitochondrial genes and normalized using the SCTransform function. Dimensionality reduction was performed using PCA, and graph-based clustering was performed using FindNeighbors and FindClusters functions with a resolution of 0.5 for clustering granularity. Visualization of the clusters in low-dimensional space was performed using nonlinear dimensionality reduction (UMAP). Clusters were grouped by the cell-type annotations provided by the metadata of the single-cell dataset. The second dataset used for comparative analysis was extracted from single-cell spatial reconstruction data. Differential gene expression data between layers of zonation were compared with markers for pericentral or periportal zonation in our dataset using R.

#### Pathway analysis.

Functional enrichment analysis of marker genes of clusters was performed using g:Profiler2 (v0.1.0). For the analysis, we extracted the gene symbols of each cluster and stored them in a list. The function “gost” of the g:Profiler2 package was then used to perform gene set enrichment analysis on input marker gene lists. In short, the function maps genes to known functional information sources and detects statistically significantly enriched terms. Since our data consist of murine liver sections, the organism was set to *Mus musculus*, and the source was set to GO biological processes.

#### Cell-cell communication.

CellChat is designed to comprehensively analyze ST data to investigate complex biological processes within an undissociated tissue.

#### Metabolic pathway analysis.

To analyze metabolic pathway activity at the single-cell level, we integrated scRNA-seq data with curated metabolic pathway databases. Using a described approach (43), which enables the reconstruction of cell type–specific metabolic profiles and allows for the comparison of metabolic activities across different cell populations, we calculated pathway activity scores by aggregating the expression of genes associated with specific metabolic pathways.

### Mass spectrometry

#### Sample preparation.

KCs (3 *×* 10^6^ cells) from both sham and I/R mice were cultured for 4 hours before harvesting. The extraction of the metabolites was performed as previously described (71). The medium was aspirated, and the cells were washed twice with liquid chromatography/mass spectrometry–grade (LC-MS–grade) water before lysing of the cells. The metabolites were extracted using a cold 80% methanol/water mixture and resuspended in 50% methanol/water mixture for further analysis using LC-MS/MS. A selected reaction monitoring (SRM) LC-MS/MS method with positive and negative ion polarity switching on a Xevo TQ-S mass spectrometer (Waters) was used for the analysis. Peak areas integrated using MassLynx 4.1 (Waters Inc.) were normalized to the respective protein concentrations.

#### Sample processing.

Untargeted analysis was performed on a Q Exactive Plus Orbitrap (Thermo Fisher Scientific) with HPLC separation on a Poroshell 120 SB-C18 column (Agilent Technologies) (2 × 100 mm, 2.7 μm particle size) with a WPS-3000 LC system (ThermoFisher Scientific). The gradient consisted of solvent A, H_2_O with 0.1 % formic acid, and solvent B, MeOH with 0.1% formic acid, at a 200 μL/min flow rate, starting at 5% solvent B with a linear ramp to 95% B at 15 minutes, holding at 95% B for 1 minute, and returning to 5% B at 17 minutes with equilibration of 5% B until 25.5 μL was injected for each sample, and the top 5 ions were selected for data-dependent analysis with a 15-second exclusion window.

#### Data processing.

For feature selection in the untargeted results analysis, including database comparison and statistical processing, samples were analyzed in Progenesis QI, (Waters) and the pooled sample runs were selected for feature alignment. The resultant peak areas were subjected to relative quantitation analyses with MetaboAnalyst 5.0 (McGill University, Montreal, Quebec, Canada). Further, PCA and pathway impact analysis and generation of heatmaps were performed using MetaboAnalyst 5.0 software.

#### Neutrophil and KC isolation and Western blotting.

Hepatic neutrophils of KCs were sorted by a BD Symphony S6 SE 6-way sorter. Whole-cell protein lysates from liver were used for Western blotting. Membranes were incubated using thrombospondin-1 (1:1,000; Cell Signaling Technology, catalog 37879, clone D7E5F), CD36 (1:1,000; Abcam, catalog ab64014), and actin (1:1,000; Cell Signaling Technology, catalog 8457S, clone D6A8) as an internal control.

#### ELISA.

TSP-1, IL-6, and myeloperoxidase (MPO) were measured in the serum using ELISA (Abcam, ab317551, for TSP-1; R&D Systems, DY406-05, for IL-6; Hycult, HK210, for MPO) according to the manufacturers’ instructions.

### Statistics

The data presented in the figures are mean ± SEM. Group comparisons were performed using analysis of variance and 2-tailed Student’s *t* test (GraphPad Prism version 8). *P* < 0.05 was considered statistically significant.

### Study approval

Animal protocols were approved by the Institutional Animal Care and Use Committee of Feinstein Institutes for Medical Research (protocol 2022-020) and performed in adherence with National Institutes of Health guidelines for use of laboratory animals.

### Data availability

The single-cell RNA-sequencing datasets associated with this study have been deposited in the NCBI Gene Expression Omnibus (GEO) under accession numbers GSE315482 and GSE173429. All other data supporting the findings of this study are available from the corresponding author upon reasonable request. This study did not generate original software or algorithms. Single-cell RNA-sequencing data were analyzed using Seurat (version 4.4), whereas spatial transcriptomic data were analyzed using publicly available software and previously published workflows, including spatial niche analysis and MISTy. The software versions, source repositories, analytical procedures, and parameters are described in the Methods and Supplementary Methods. This study did not generate new unique reagents. Additional details regarding experimental procedures, sample processing, sequencing, and computational analyses are provided in the Supplementary Methods section of the Supplementary Information.

## Author contributions

HZ and HH conceived and designed the study. HZ, XH, KC, AEV, MD, and HH developed methodology. XH, YZ, TY, CS, AK, VB, AO, DL, XG, MD, and HH acquired data (provided animals, acquired and managed patients, provided facilities, etc.). HZ, XH, PJ, YZ, AK, VB, PSL, JMC, CS, and HH analyzed and interpreted data (e.g., statistical analysis, biostatistics, computational analysis). HZ, XH, GW, MD, JMC, and HH wrote, reviewed, and/or revised the manuscript. XH, CS, KC, AEV, AT, LM, and MD provided administrative, technical, or material support (e.g., reporting or organizing data, constructing databases). HH supervised the study.

## Conflict of interest

The authors have declared that no conflict of interest exists.

### Financial support

This work is the result of NIH funding, in whole or in part, and is subject to the NIH Public Access Policy. Through acceptance of this federal funding, the NIH has been given a right to make the work publicly available in PubMed Central.

National Institute of General Medical Sciences (R01 GM137203 and R35 GM163879, to HH).

Northwell Health Research Bridge Program award (to HH).

National Institute of Allergy and Infectious Diseases (R01 AI152044 and R21AI182698, to MD).

National Cancer Institute (R01 CA214865, to AT).

State funding within the UVA Comprehensive Cancer Center (IDEA-Cancer pilot award and Cancer Therapeutics [CRX] pilot award, to HZ).

Office of the Director, National Institutes of Health (S10 OD036216, to flow cytometry core, Feinstein Institutes for Medical Research).

## Supplementary Material

Supplemental data

Unedited blot and gel images

Supporting data values

## Version 1 09/22/2026

Electronic publication
