# Supplementary material for: Integrated single-cell multiomics reveals neutrophil-driven immune-metabolic reprogramming of Kupffer cells via thrombospondin-1/CD36 after surgical stress
Source: JCI Insight. 2026 Sep 22;11(18):e203495. doi: 10.1172/jci.insight.203495 (PMC13596724; doi:10.1172/jci.insight.203495)
Supplement: Supplementary file 1 [file jciinsight-11-203495-s289.pdf]

Supplementary Figures

Supplementary Fig. 1: Hepatic injury and immune cell dynamics following liver I/R.

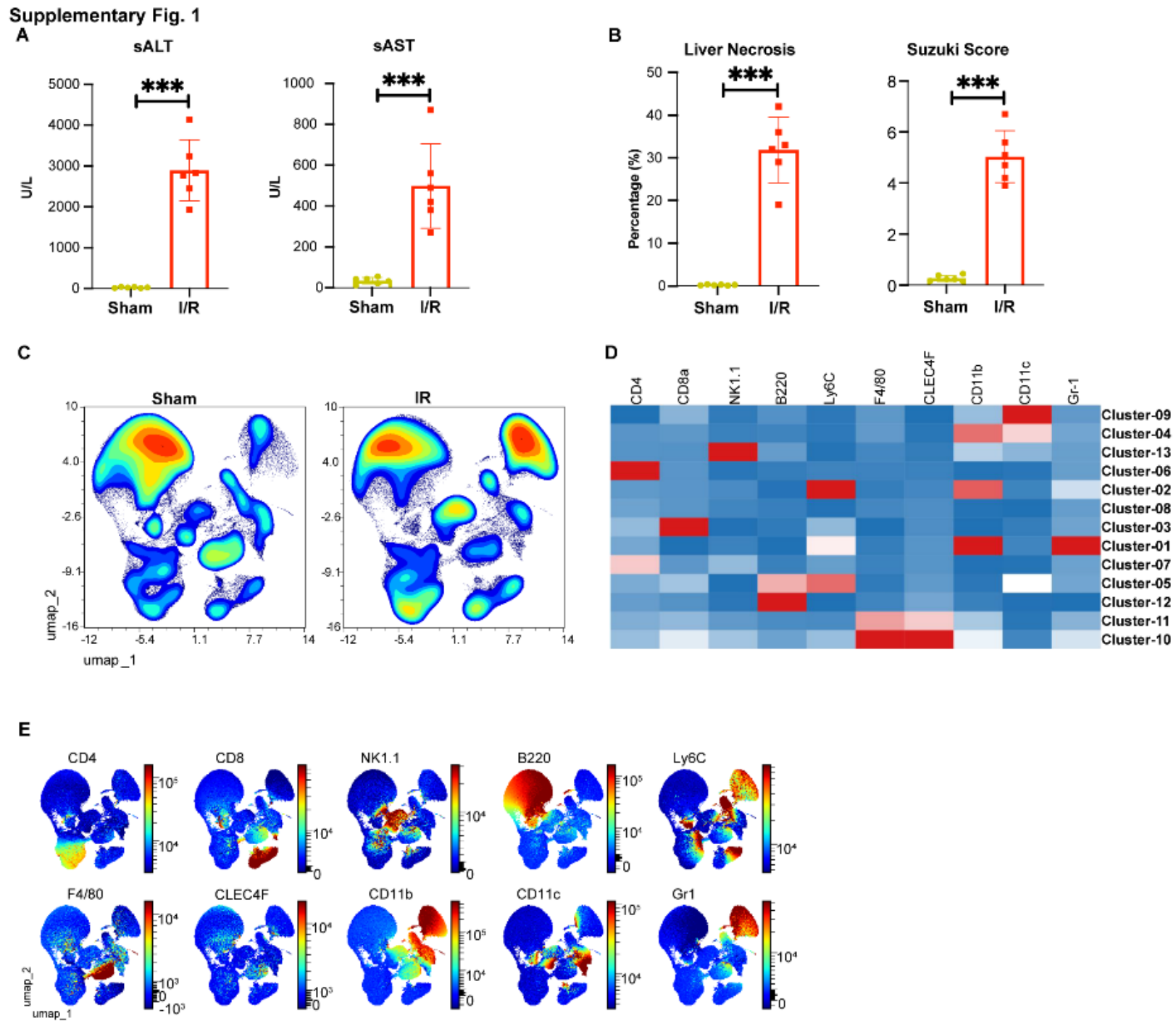

(**A**) Serum alanine aminotransferase (sALT) and aspartate transaminase (sAST) levels were quantified in sham and I/R mice. (**B**) Quantification of liver necrosis percentage and Suzuki score in sham and I/R liver. (**C**) UMAP plots of FlowSOM data showing distinct immune cell populations in sham and I/R livers. (**D**) Heatmap showing the relative marker expression in identified immune cell clusters based on FlowSOM analysis. (**E**) UMAP plots depicting the distribution of markers for identification of immune cell populations.

Supplementary Fig. 2: scRNA-seq and immune cell distribution before and after liver I/R.

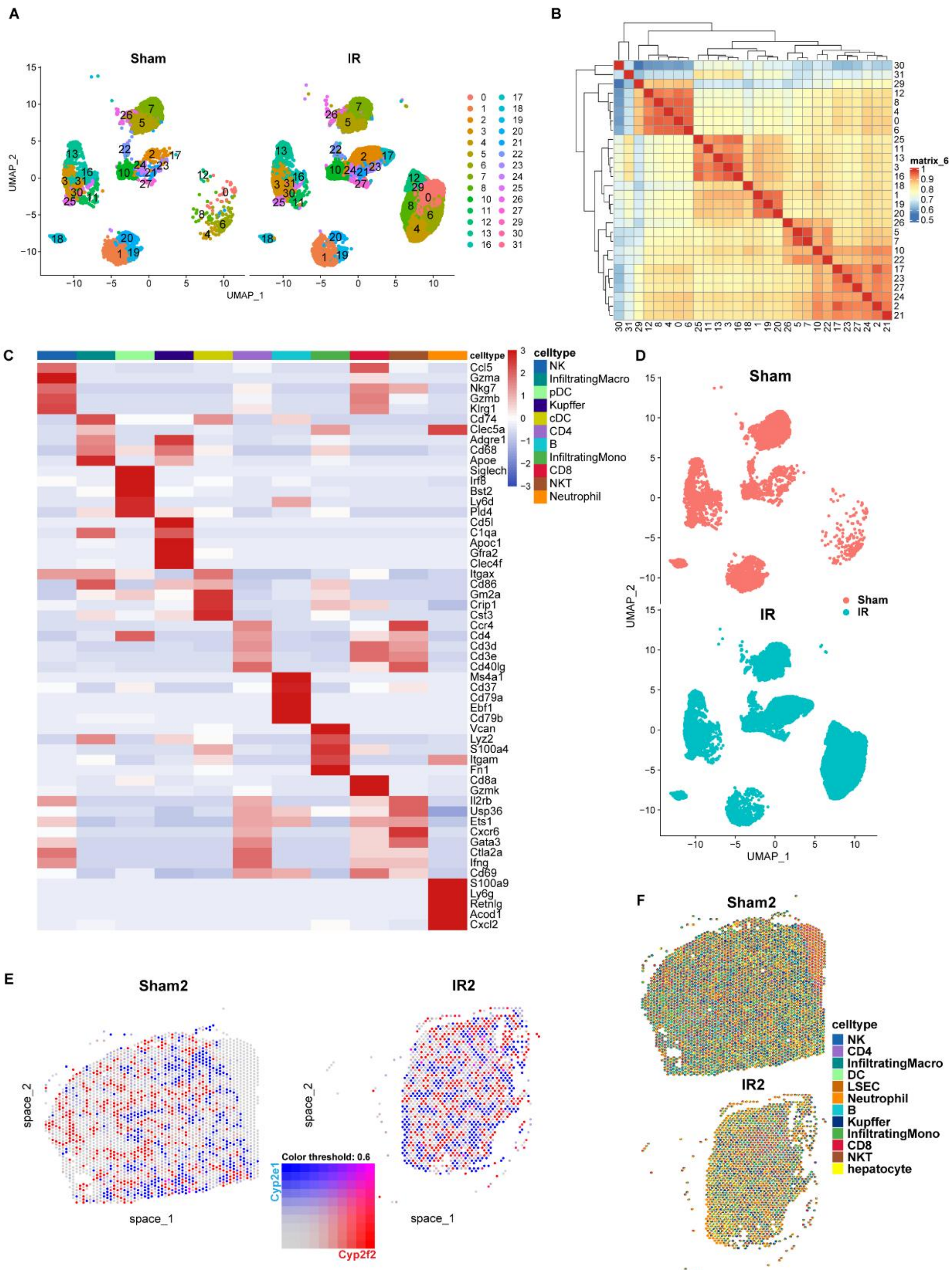

(**A**) UMAP of scRNA-seq data showing CD45^{+} immune cell types extracted from the global UMAP space in sham and I/R livers. (**B**) Heatmap of pairwise correlations between 32 discrete clusters identified in the hepatic scRNA-seq dataset. (**C**) Heatmap showing expression of key feature genes for different immune cell types. (**D**) UMAP visualization of CD45^{+} immune cells comparing sham (left, red) and I/R (right, blue) livers. (**E**) Identification of liver zonation in spatial transcriptomics specimens based on expression of *Cyp2f2* and *Cyp2e1* of Sham2 and I/R2 livers. (**F**) Spatial scatter-pie plot in which each spot is represented as a pie chart indicating the predicted proportion of constituent cell types of Sham2 and I/R2 livers.

Supplementary Fig. 3: Characterization of CD4⁺ T cells after liver I/R.

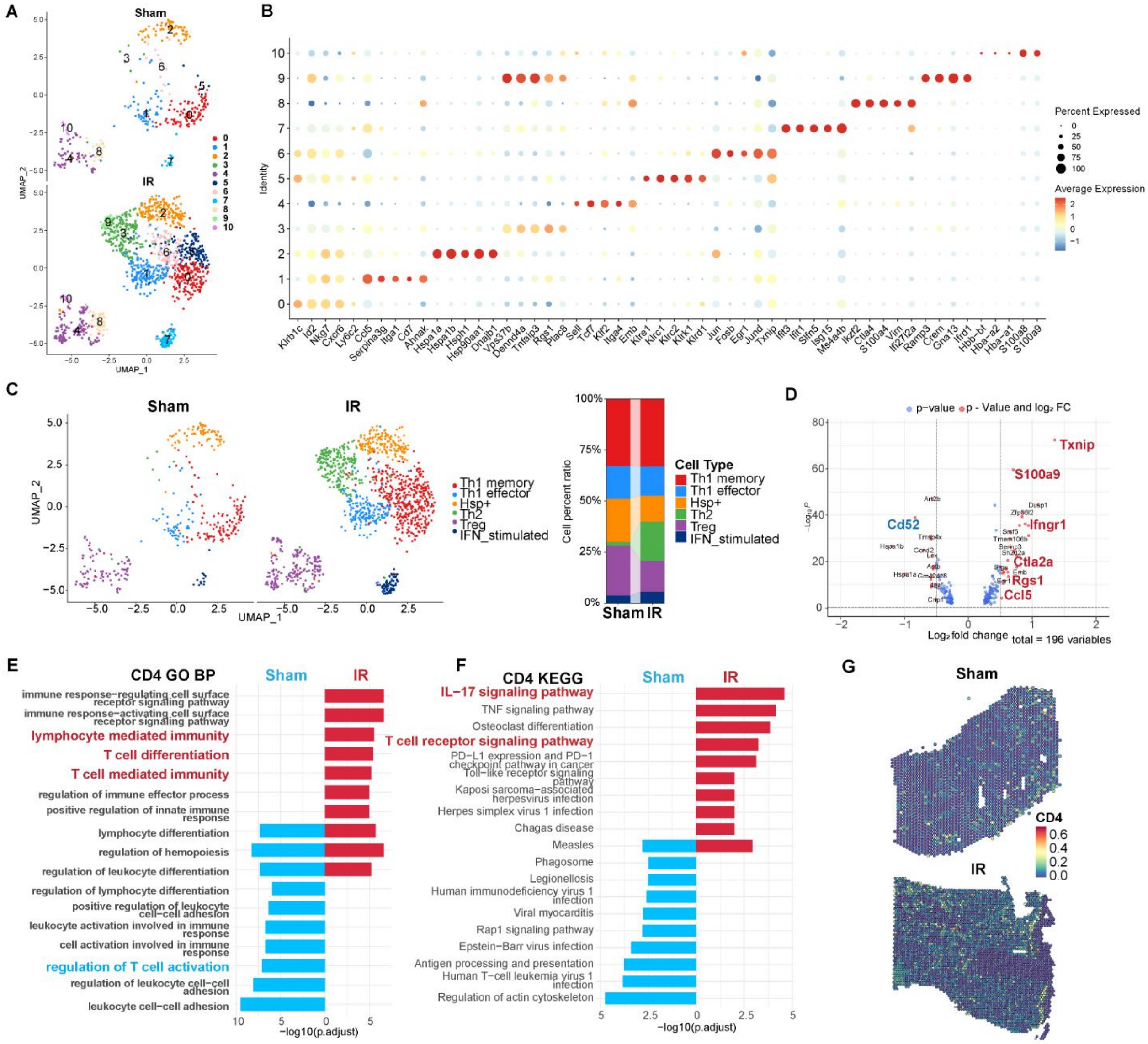

(A) UMAP of CD4⁺ T cell subclusters colored and numbered by scRNA-seq clusters. (**B**) Dot plot showing the expression of key DEGs across CD4⁺ T cell subclusters. (**C**) UMAP plots showing the distribution of CD4⁺ T-cell subtypes. (**D**) Volcano plot illustrating differentially expressed genes (DEGs) in I/R-associated CD4⁺ T cells compared with sham. (**E**) GO Biological Process pathway enrichment analysis of DEGs in I/R-associated CD4⁺ T cells compared with sham. (**F**) KEGG pathway enrichment analysis of DEGs in I/R-associated CD4⁺ T cells versus sham. (**J**) Spatial transcriptomics analysis showing the distribution and localization of CD4⁺ T cells in sham and I/R mouse livers.

Supplementary Fig. 4: Characterization of CD8⁺ T cells and NKT cell after liver I/R.

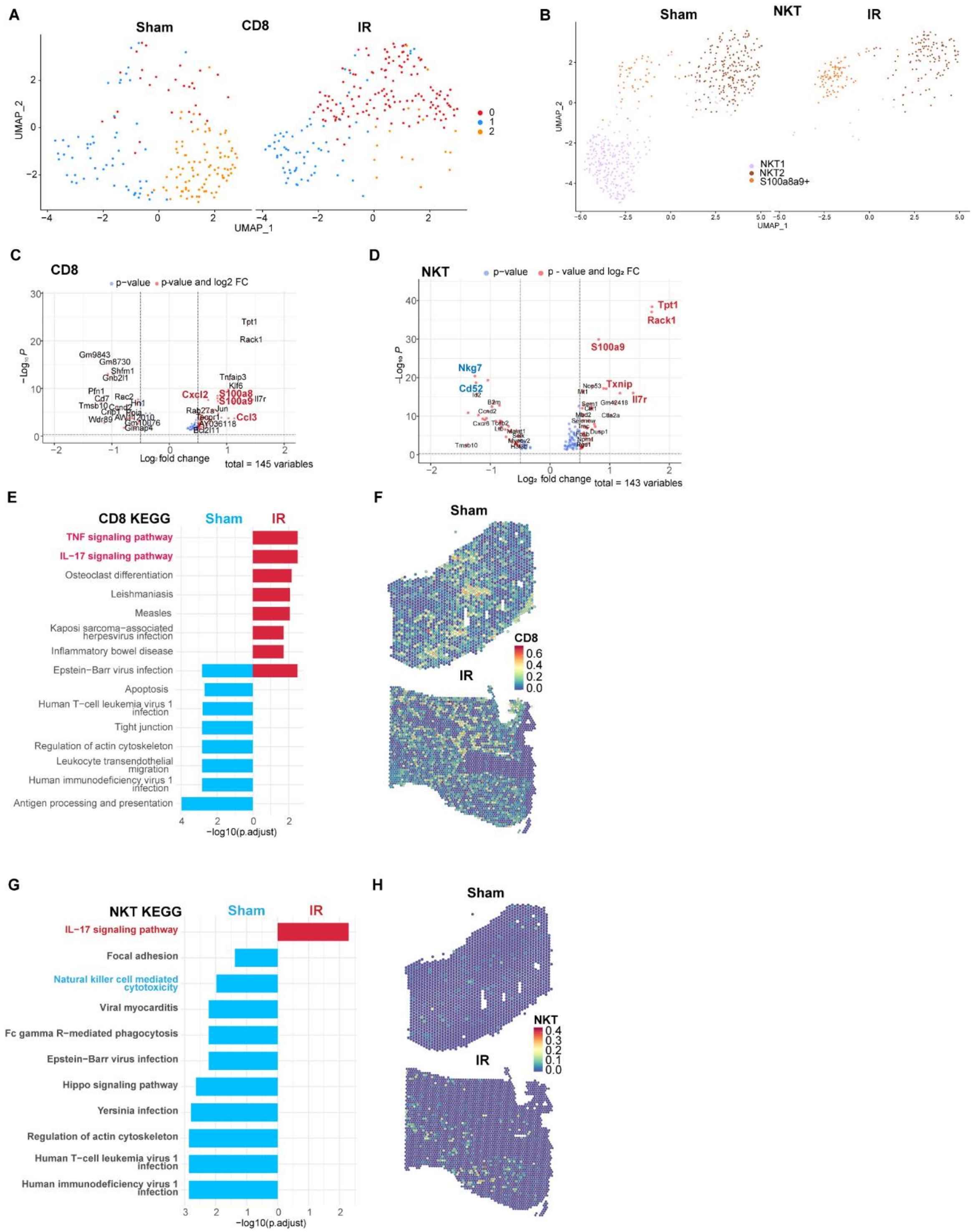

(A) UMAP of CD8⁺ T cell subclusters colored and numbered by scRNA-seq clusters. (**B**) UMAP of NKT cell subclusters colored and numbered by scRNA-seq clusters. (**C**) Volcano plot showing DEGs in I/R-associated CD8⁺ T cells compared to sham. (**D**) Volcano plot showing DEGs in I/R-associated NKT cells compared to sham. (**E**) KEGG pathway enrichment analysis of DEGs in I/R-associated CD8⁺ T cells versus sham. (**F**) Spatial transcriptomics analysis of CD8⁺ T cells in sham and I/R mouse livers. (**G**) KEGG pathway enrichment analysis of DEGs in I/R-associated NKT cells versus sham. (**H**) Spatial transcriptomics analysis of NKT cells in sham and I/R mouse liver.

Supplementary Fig. 5: Characterization of B cells after liver I/R.

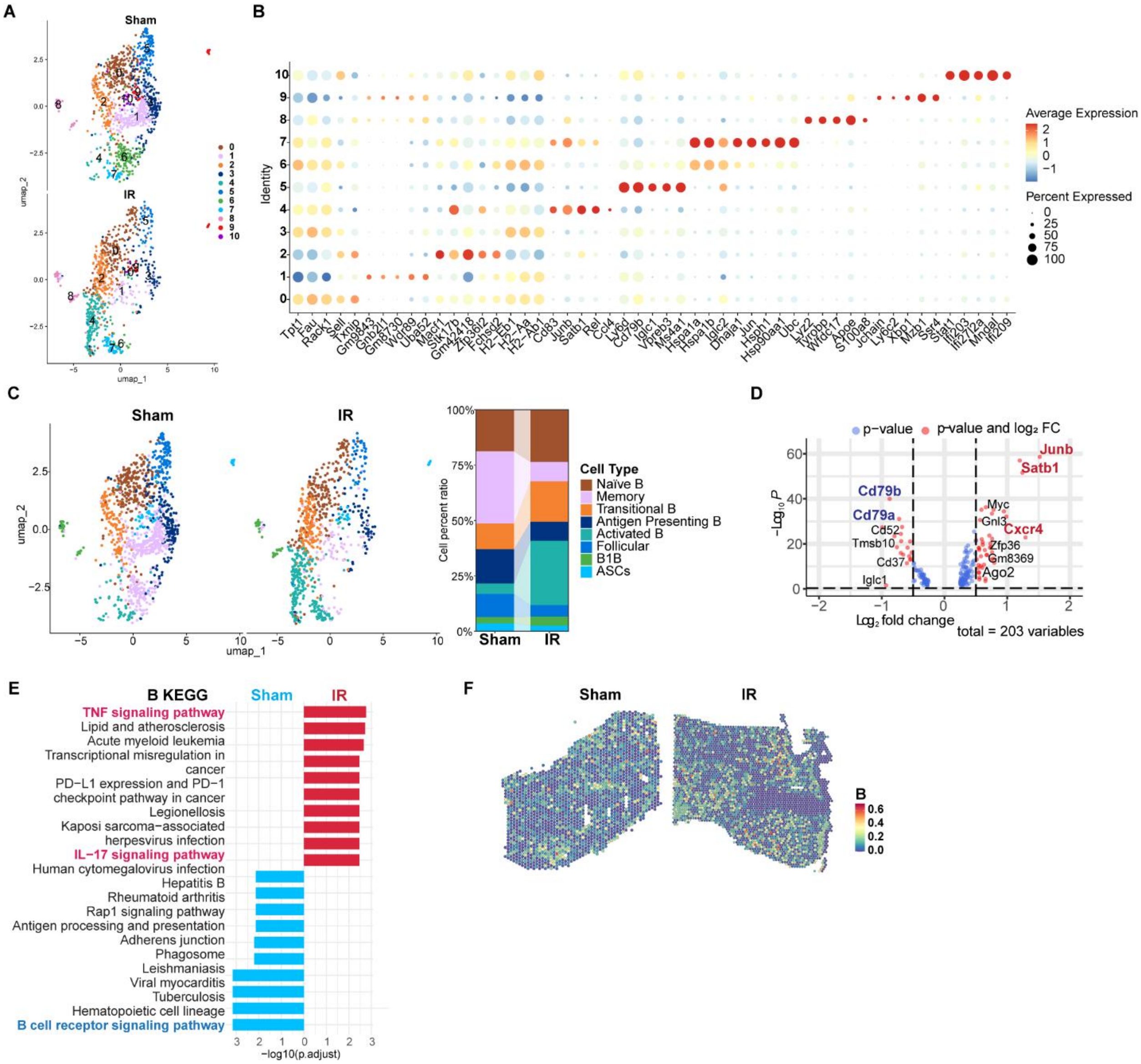

(A) UMAP of B cell subclusters colored and numbered by scRNA-seq clusters. (**B**) Dot plot showing the expression of key DEGs across the identified B cell subclusters. (**C**) UMAP plots showing the distribution of B cell subtypes. (**D**) Volcano plot showing DEGs in I/R-associated B cells compared to sham B cells. (**E**) KEGG pathway enrichment analysis of DEGs in I/R-associated B cells versus sham. (**F**) Spatial transcriptomics analysis of B cells in sham and I/R mouse livers.

Supplementary Fig. 6: Characterization of NK cells after liver I/R.

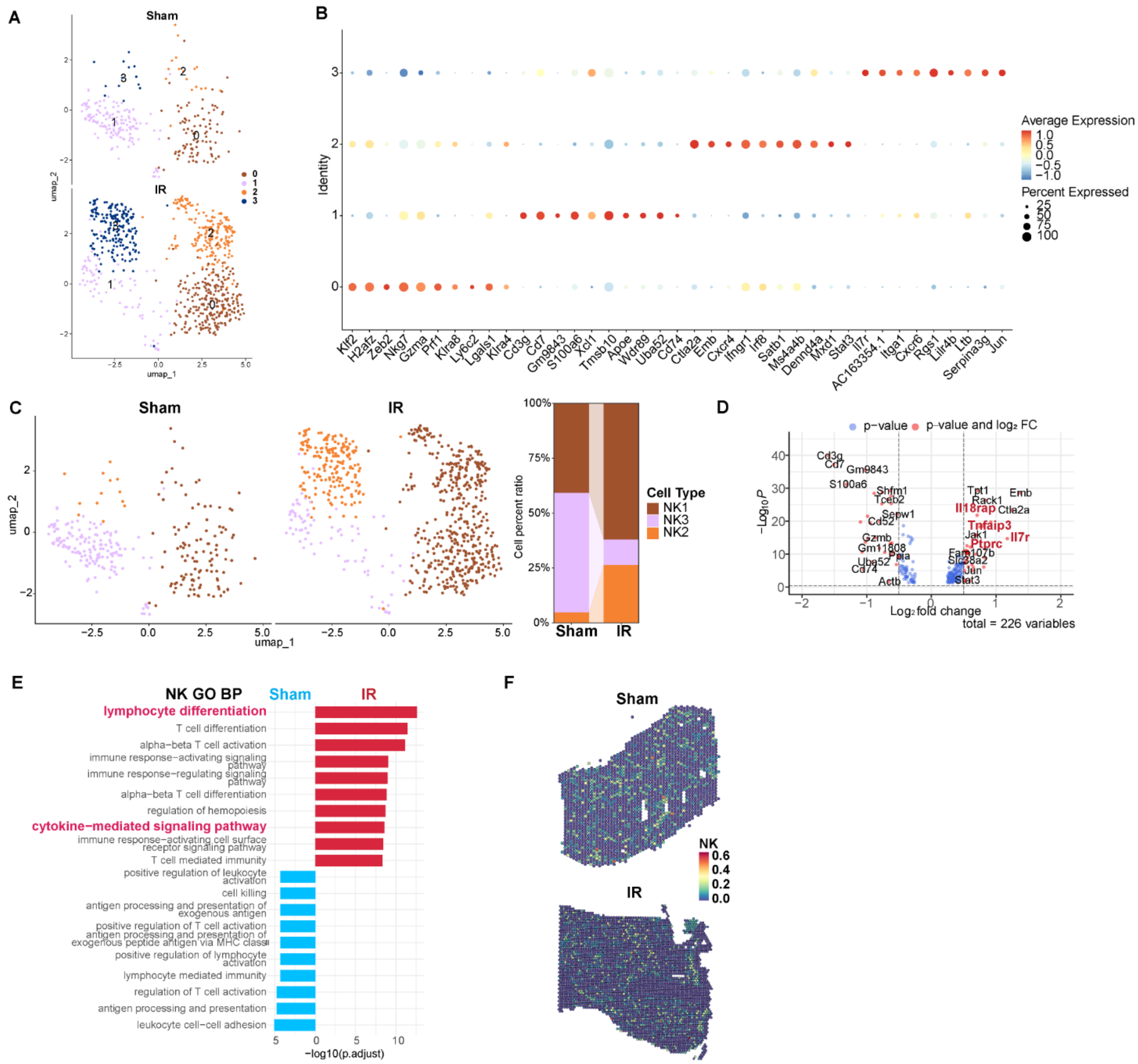

(A) UMAP of NK cell subclusters colored and numbered by scRNA-seq clusters. (**B**) Dot plot showing the expression of key DEGs across the identified NK cell subclusters. (**C**) UMAP plots showing the distribution of NK cell subtypes. (**D**) Volcano plot showing DEGs in I/R-associated NK cells compared to sham B cells. (**E**) GO Biological Process pathway enrichment analysis of DEGs in I/R-associated NK cells versus sham. (**F**) Spatial transcriptomics analysis of NK cells in sham and I/R mouse livers.

Supplementary Fig. 7: Characterization of Dendritic cells (DCs) after liver I/R.

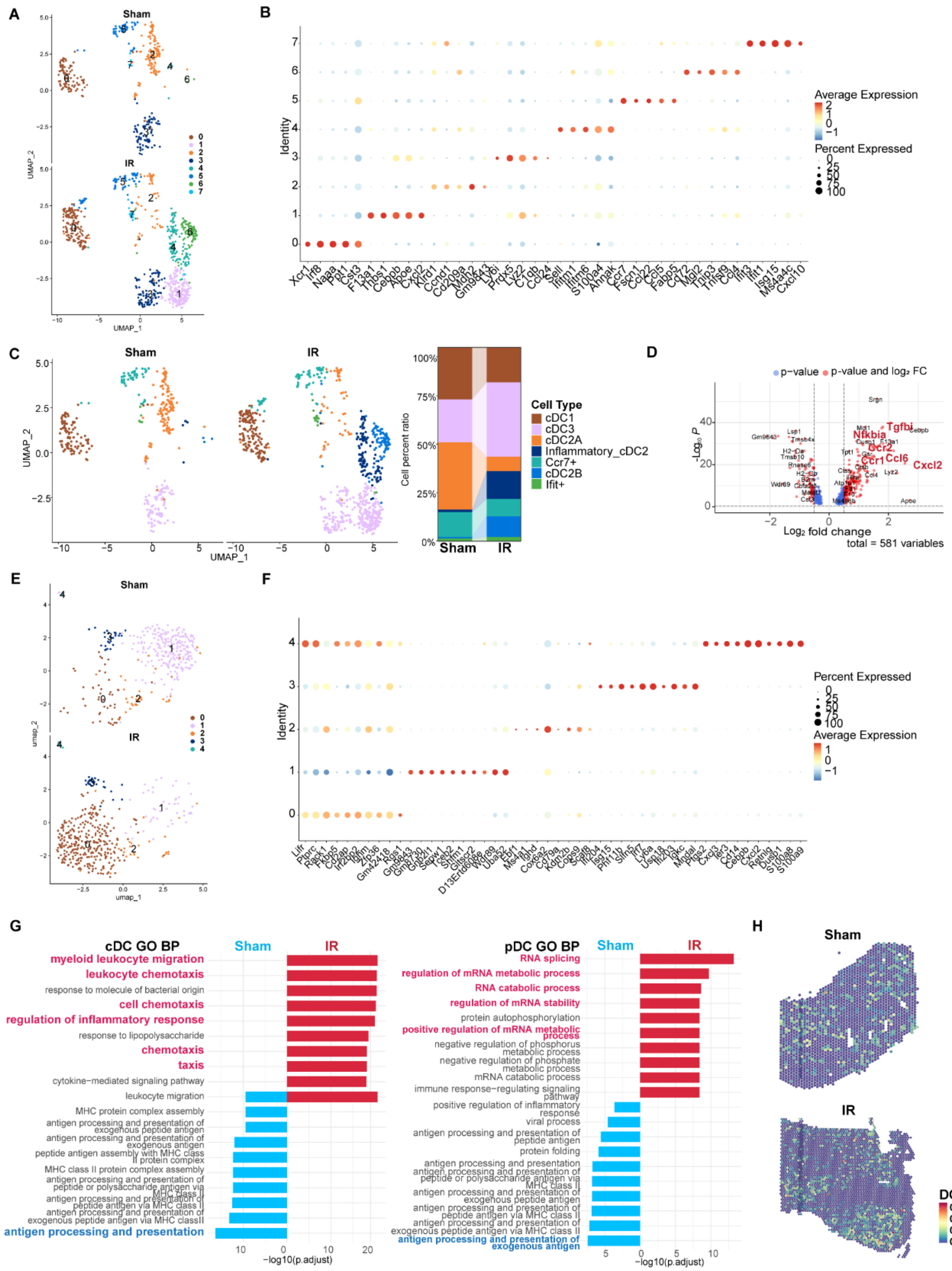

(A) UMAP of Conventional DCs (cDCs) subclusters colored and numbered by scRNA-seq clusters. (**B**) Dot plot showing the expression of key DEGs across the identified cDCs cell subclusters. (**C**) UMAP plots showing the distribution of cDCs cell subtypes. (**D**) Volcano plot showing DEGs in I/R-associated cDCs cells compared to sham B cells. (**E**) UMAP of Plasmacytoid DCs (pDCs) subclusters colored and numbered by scRNA-seq clusters. (**F**) Dot plot showing the expression of key DEGs across the identified pDCs cell subclusters. (**G**) GO Biological Process pathway enrichment analysis of DEGs in I/R-associated cDCs (left), and pDCs (right) versus sham. (**H**) Spatial transcriptomics analysis of broad DCs in sham and I/R mouse livers.

Supplementary Fig. 8: Characterization of infiltrating monocytes and infiltrating macrophages after liver I/R.

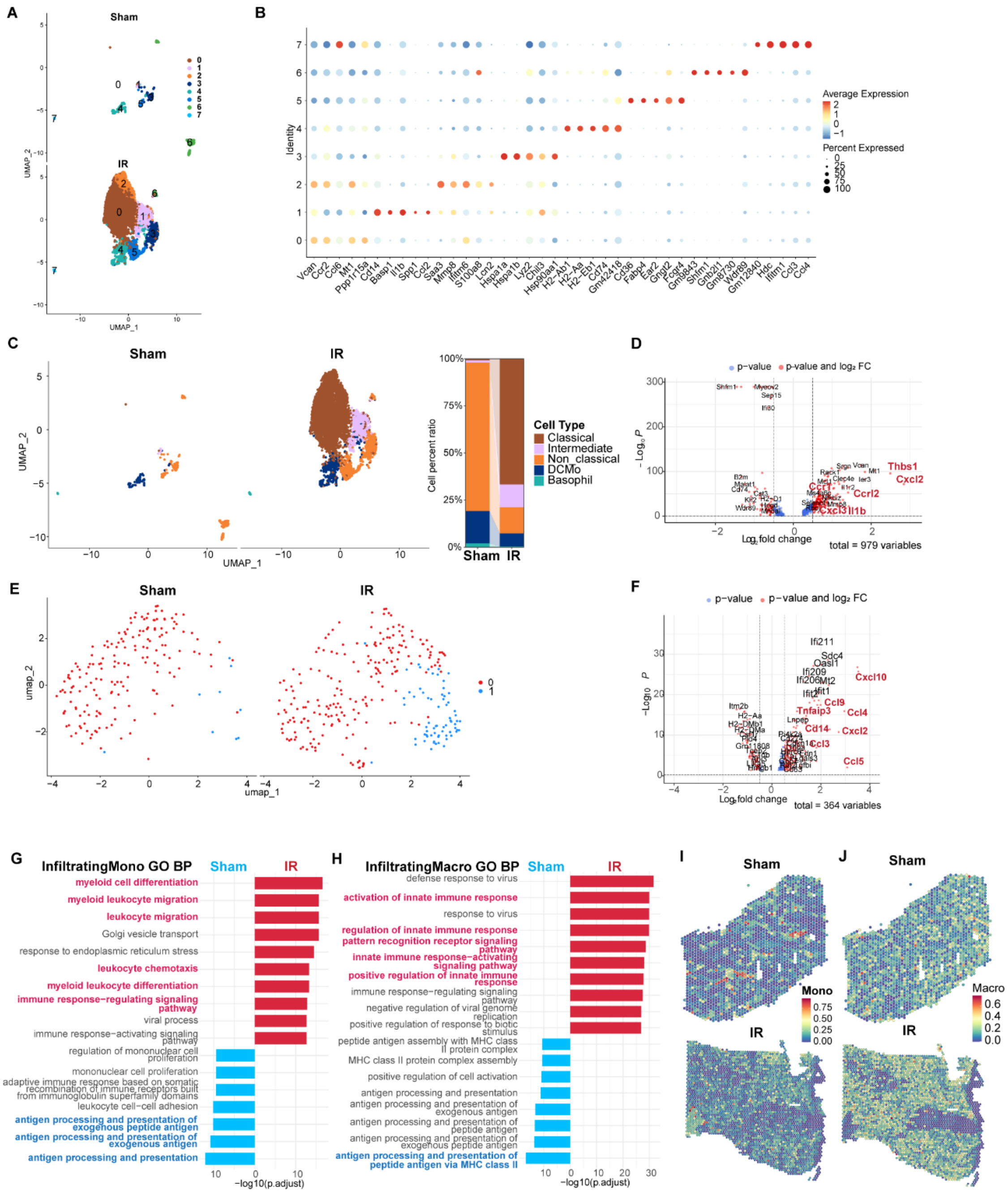

(A) UMAP of Conventional infiltrating monocyte subclusters colored and numbered by scRNA-seq clusters. (**B**) Dot plot showing the expression of key DEGs across the identified infiltrating monocyte subclusters. (**C**) UMAP plots showing the distribution of infiltrating monocyte cell subtypes. (**D**) Volcano plot shows DEGs in I/R-associated infiltrating monocyte cells compared to sham B cells. (**E**) UMAP of infiltrating macrophage subclusters colored and numbered by scRNA-seq clusters. (**F**) Volcano plot showing DEGs in I/R-associated infiltrating macrophages compared to sham. (**G**) GO Biological Process pathway enrichment analysis of DEGs in I/R-associated infiltrating monocyte versus sham. (**H**) GO Biological Process pathway enrichment analysis of DEGs in I/R-associated infiltrating monocyte versus sham. (**I**) Spatial transcriptomics analysis of broad DCs in sham and I/R mouse livers.

Supplementary Fig. 9: Characterization of Kupffer cells and neutrophils after liver I/R.

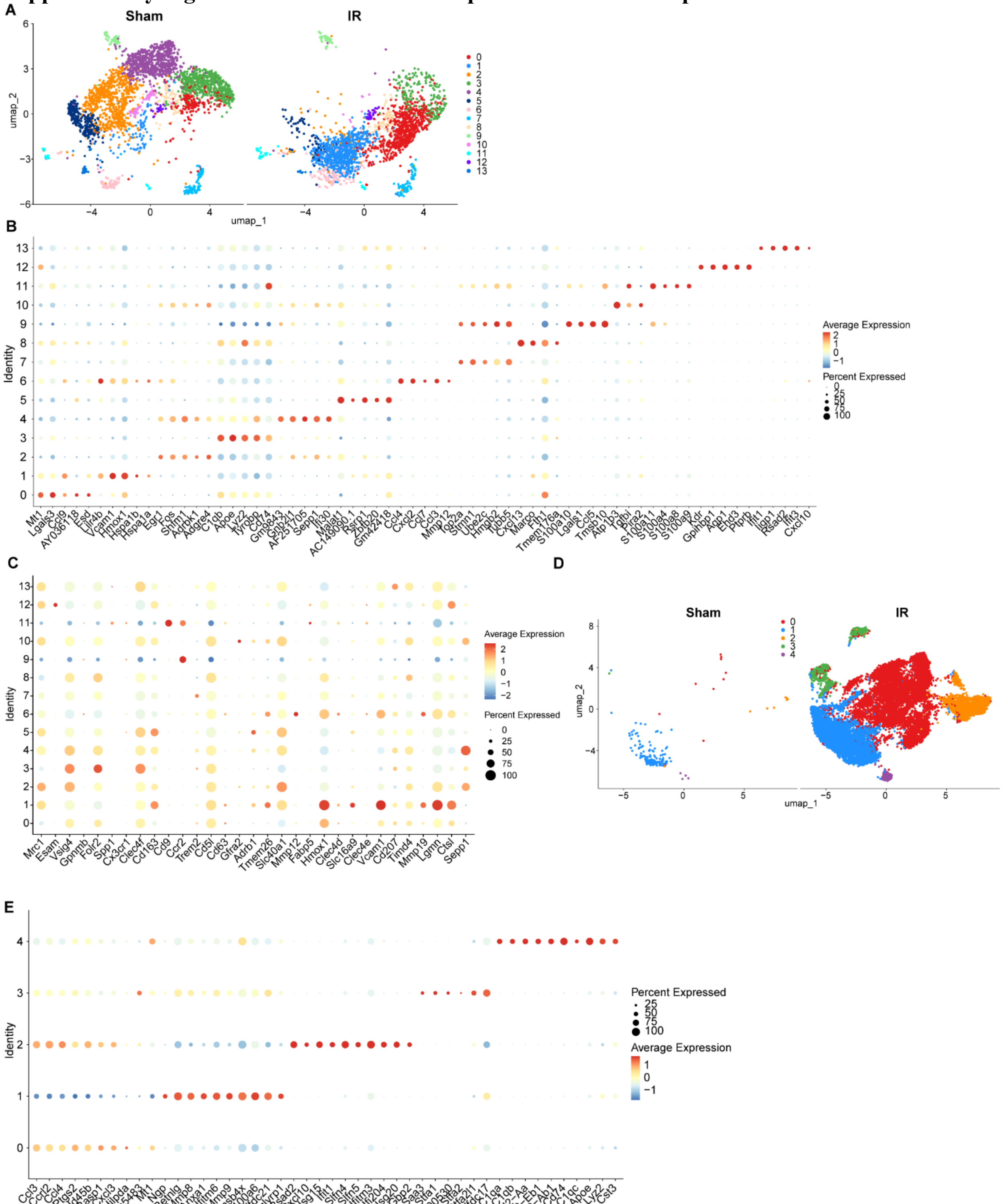

(A) UMAP of Kupffer cell subclusters colored and numbered by scRNA-seq clusters. (**B**) Dot plot showing the expression of DEGs across Kupffer cell subclusters. (**C**) Dot plot showing the expression of key feature genes across Kupffer cell subclusters for annotation of Kupffer cell subtypes. (**D**) UMAP of neutrophil subclusters colored and numbered by scRNA-seq clusters. (**E**) Dot plot showing the expression of key DEGs across neutrophil subclusters.

Supplementary Fig. 10: Interaction between neutrophils and Kupffer cells mediated by TSP-1/CD36 signaling during liver I/R.

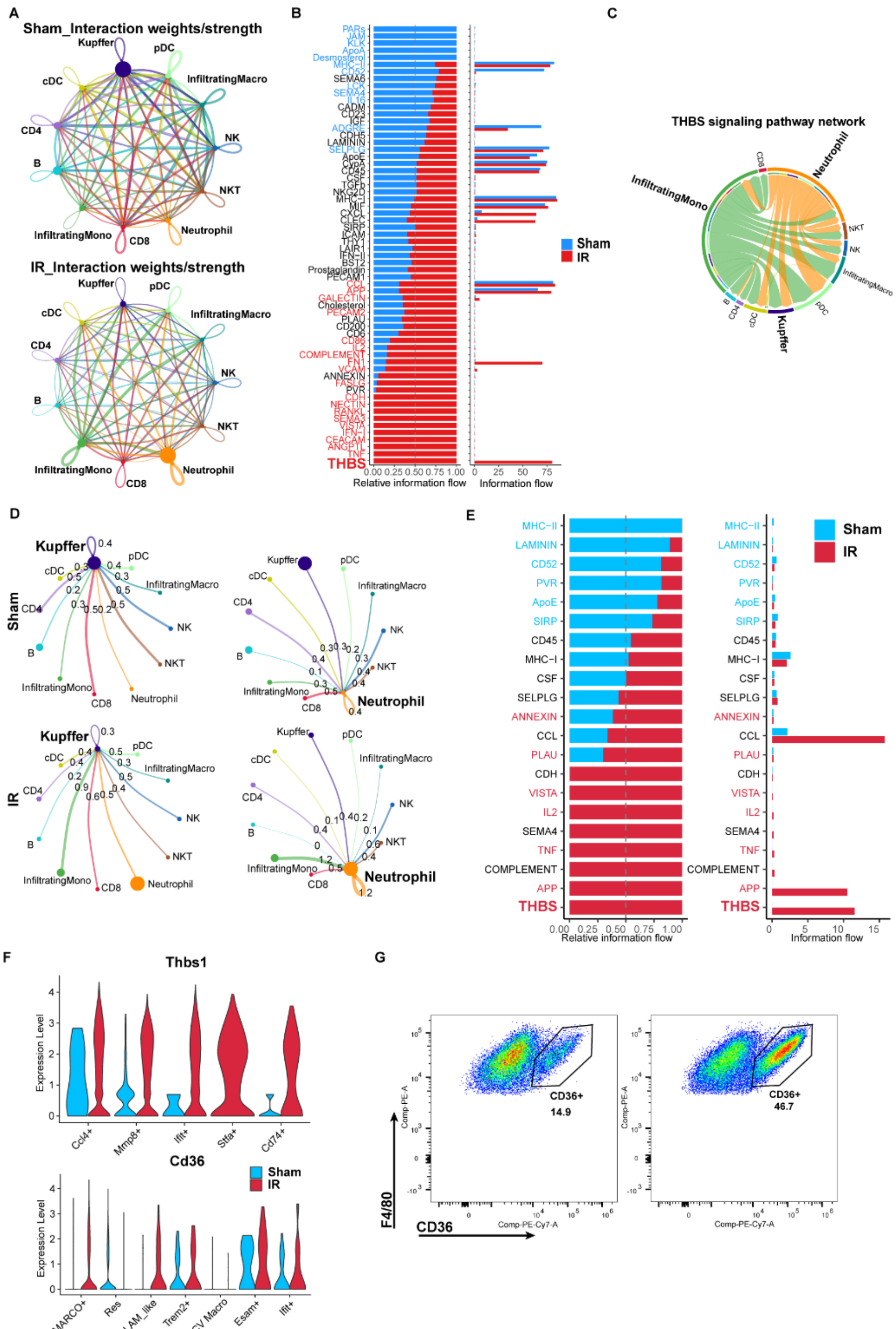

(**A**) Global cell–cell interaction analysis of hepatic immune cells in sham (left) and I/R (right) liver using CellChat analysis. (**B**) Bar graph showing the relative information flow of ligand–receptor interactions among all hepatic immune cell types before and after I/R. (**C**) Chord diagram of THBS signaling between hepatic immune cells following I/R. (**D**) Circle plot showing predicted incoming cell–cell interactions to KCs (left) and neutrophils (right) from other hepatic immune cell subsets in sham (left) and I/R (right) groups using CellChat analysis. (**E**) Bar graph showing the relative information flow of ligand–receptor interactions from neutrophils to other immune cells before and after I/R. (**F**) Violin plot showing *Thbs1* expression across neutrophil subtypes and *Cd36* expression across Kupffer cell subtypes. (**G**) Flow cytometry showing CD36^{+} KCs from sham and I/R livers.

Supplementary Fig. 11: Validation of TSP-1/CD36 signaling in human neutrophil–Kupffer cell interactions during liver transplantation.

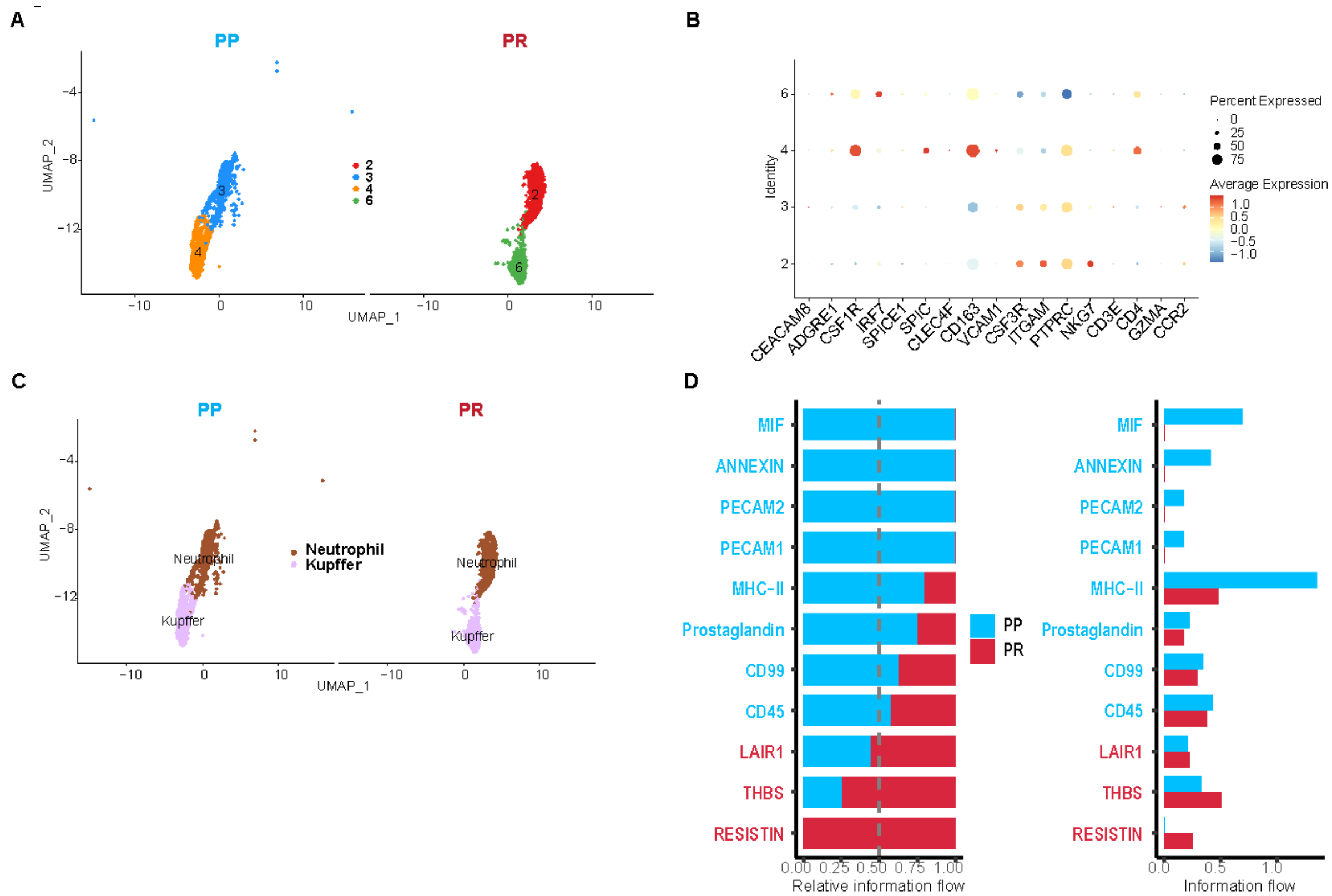

(**A**) UMAP of Kupffer cell and neutrophil subclusters colored and numbered by publicly available scRNA-seq dataset (GSE171539). (**B**) Dot plot showing the expression of key feature genes across Kupffer cell and neutrophil populations. (**C**) UMAP of Kupffer cells and neutrophils colored by the same scRNA-seq dataset (GSE171539).

Supplementary Fig. 12: Spatial transcriptomics reveal enhanced neutrophil–KC interaction after liver I/R.

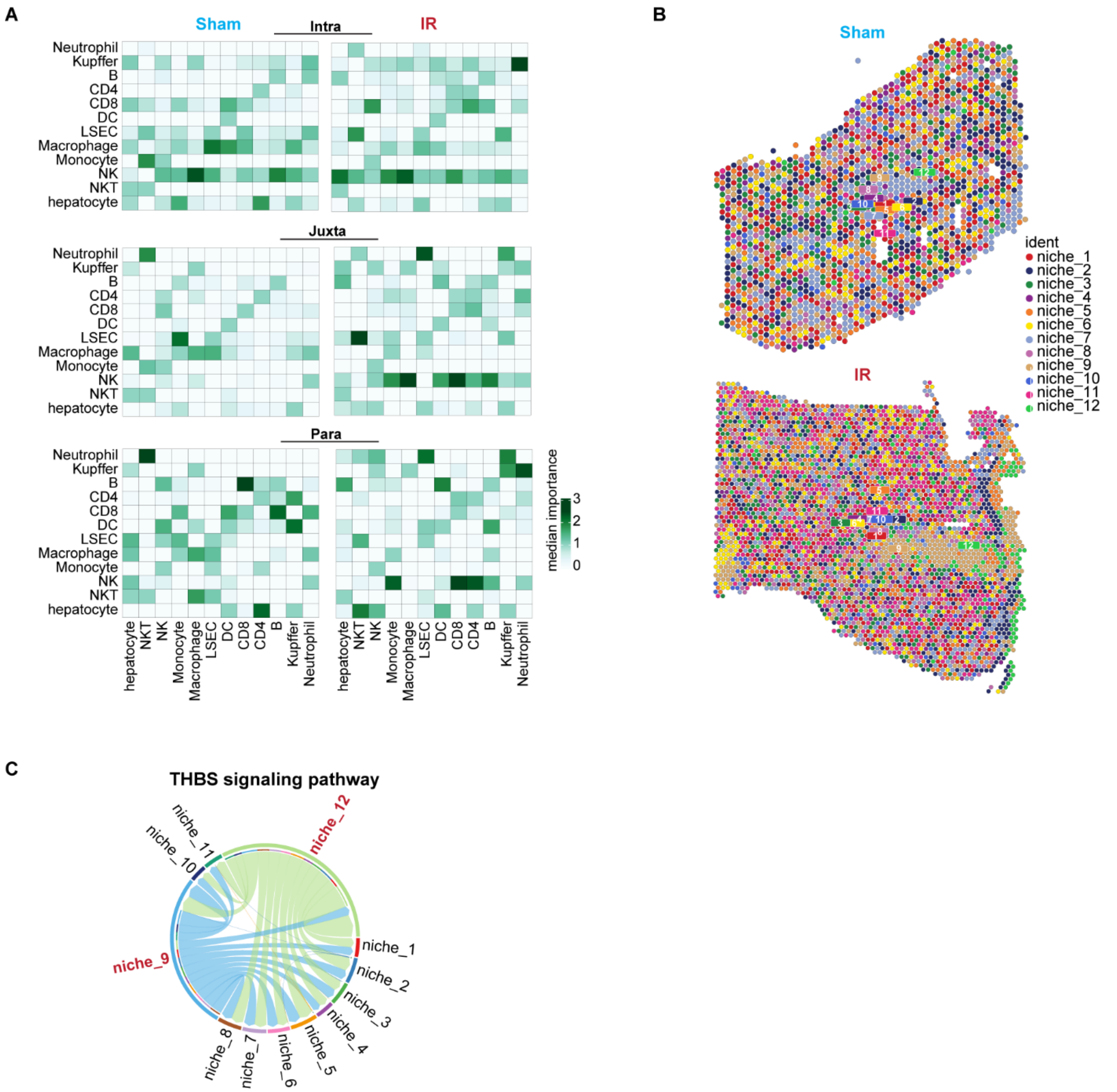

(**A**) Heatmap using MISTy to evaluate spatial dependencies between immune cell types within three neighborhood contexts: co-localization within the same spot (Intra), immediate neighborhood (Juxta), and extended neighborhood (Para). (**B**) Spatial distribution of neutrophil-dominant niches in I/R liver compared to sham. (**C**) Spatial enrichment of THBS signaling from neutrophil-dominant niches.
