# Supplementary material for: Integrated single-cell multiomics reveals neutrophil-driven immune-metabolic reprogramming of Kupffer cells via thrombospondin-1/CD36 after surgical stress
Source: JCI Insight. 2026 Sep 22;11(18):e203495. doi: 10.1172/jci.insight.203495 (PMC13596724; doi:10.1172/jci.insight.203495)

Full unedited blot/gel for Figure 6E upper panel

TSP-1

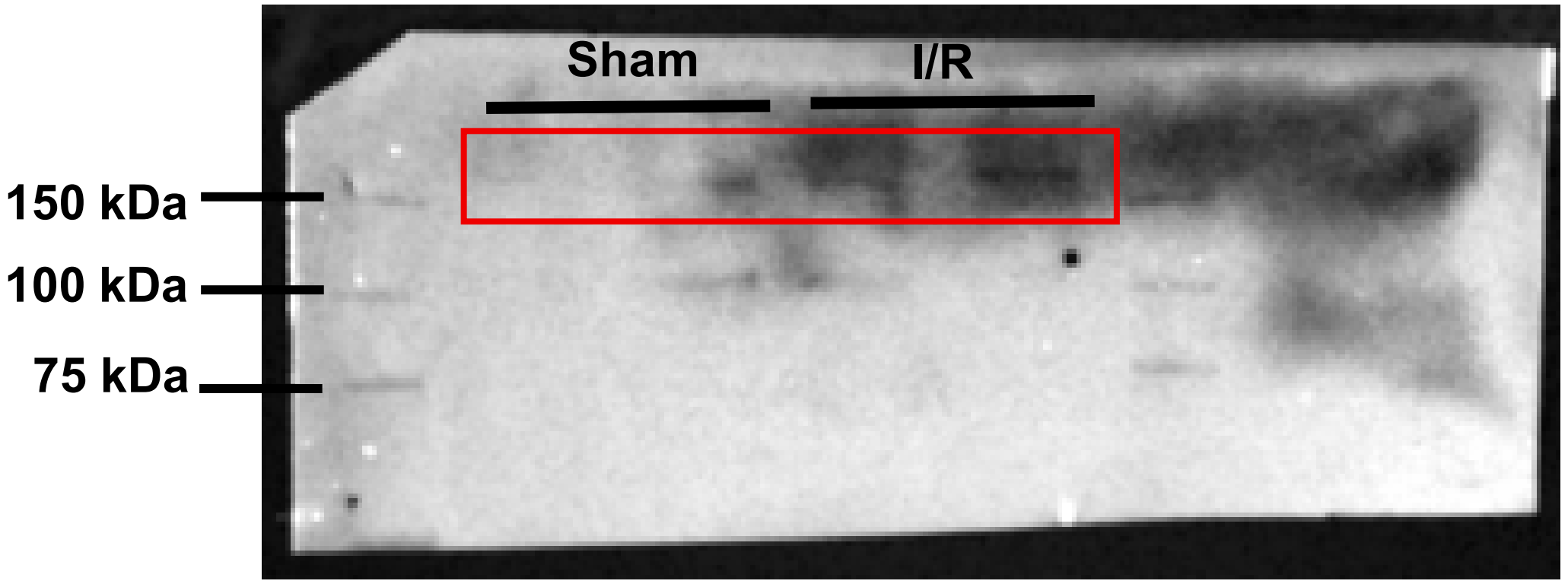

β-actin

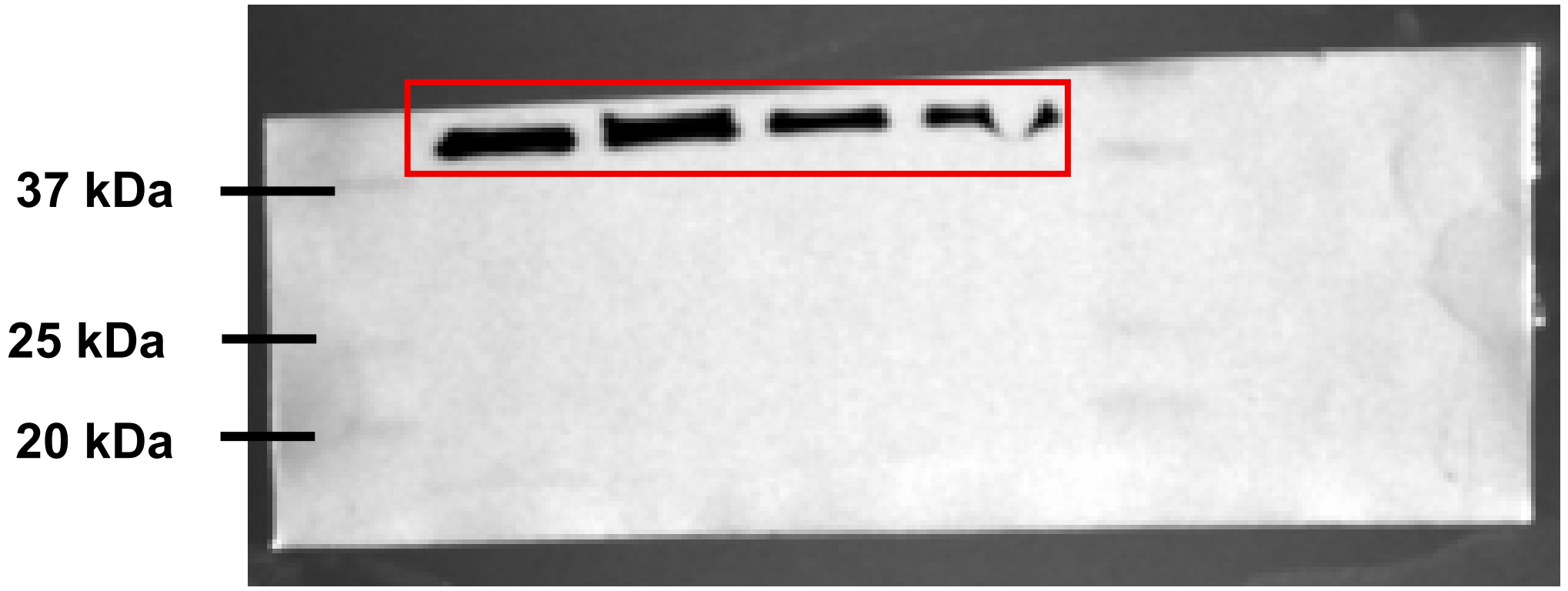

Full unedited blot/gel for Figure 6E bottom panel

CD36

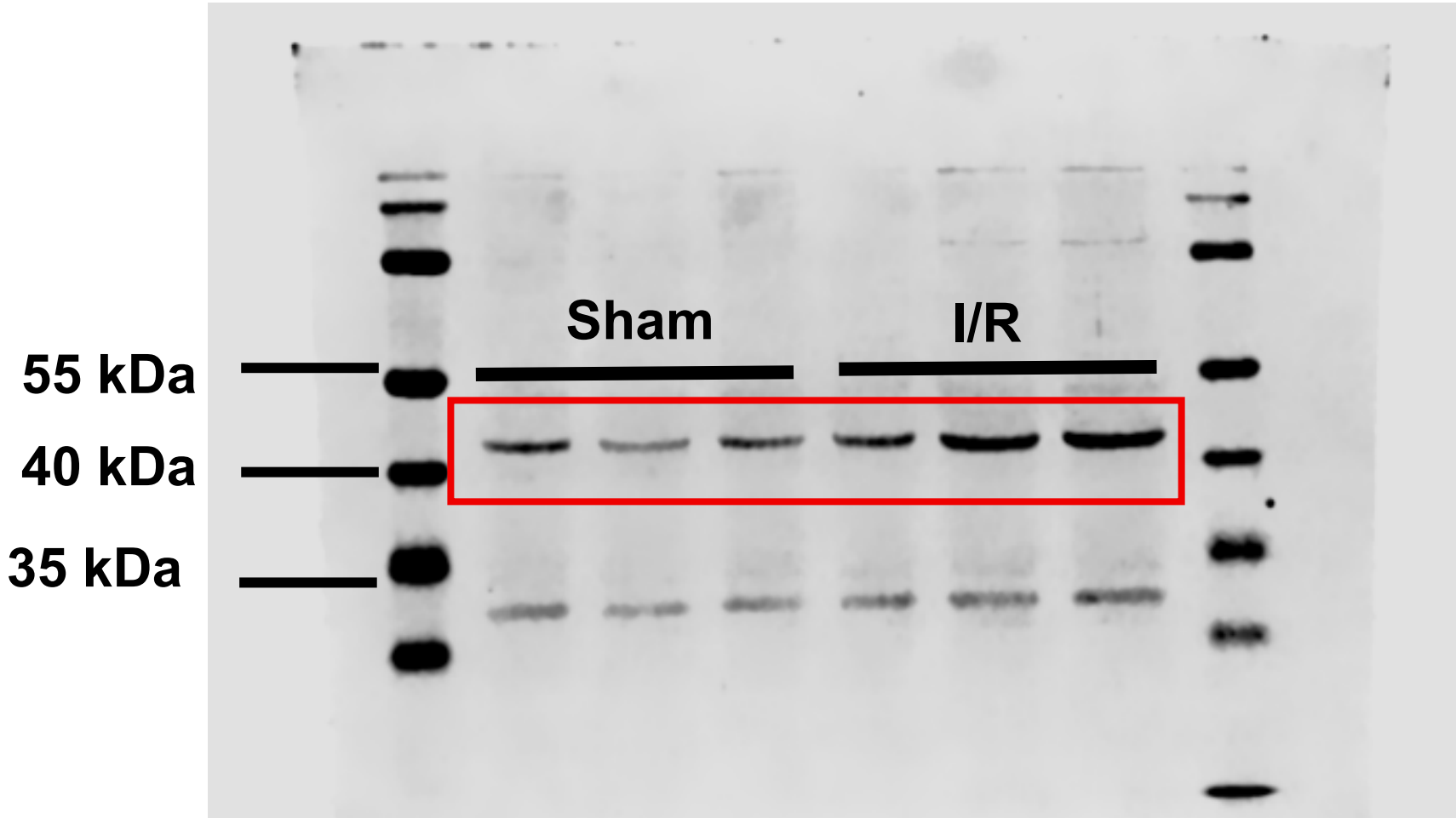

β-actin

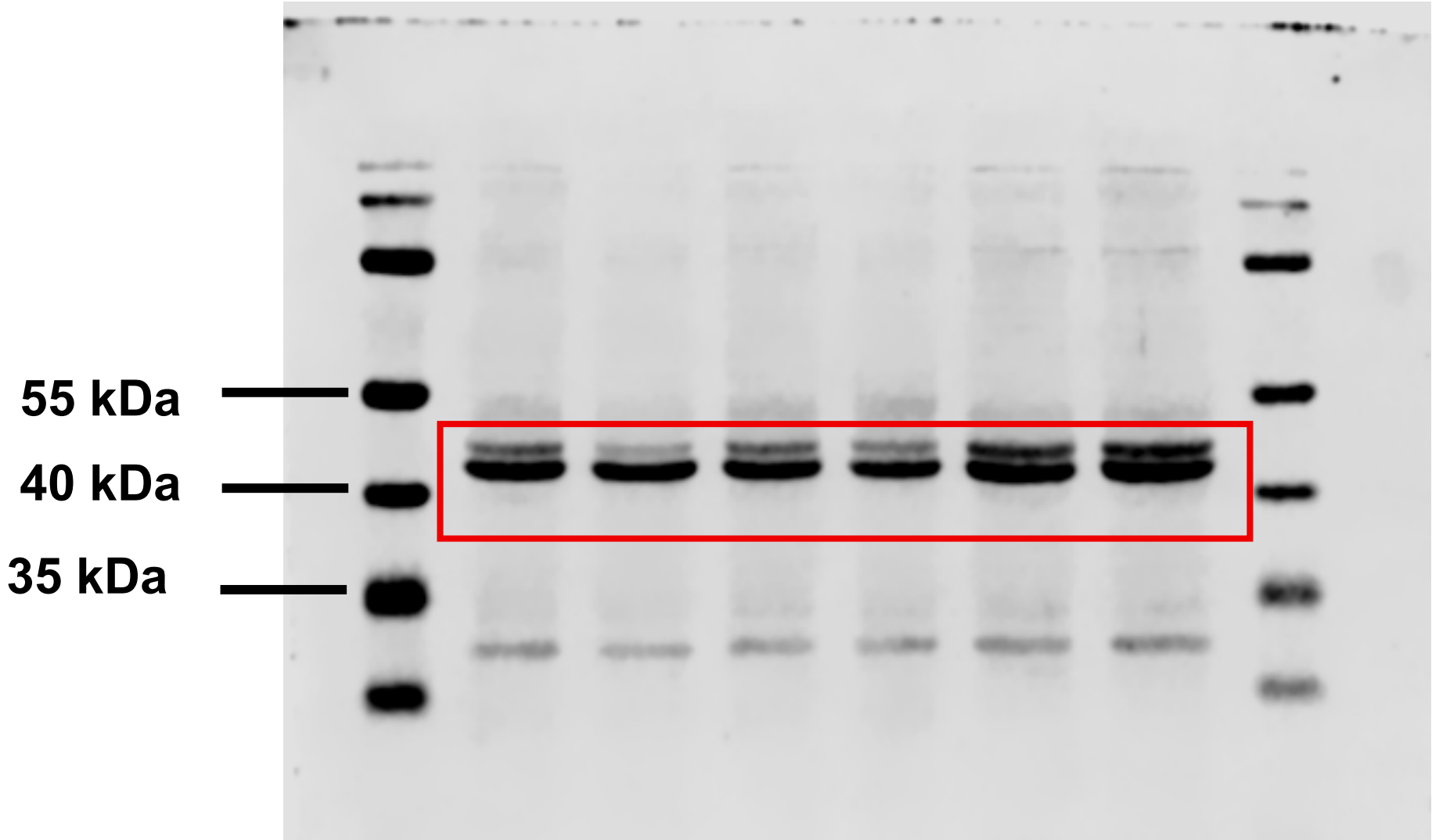

Supplement: Supplementary file 2 [file jciinsight-11-203495-s290.pdf]
